# Resveratrol and the neuroinflammation axis in Alzheimer’s disease, Parkinson’s disease, multiple sclerosis, and cerebral ischemia

**DOI:** 10.3389/fimmu.2026.1758356

**Published:** 2026-04-21

**Authors:** Haoyu Wang, Fei Li, Haifan Wang, Zenan Tian, Hong Fan, Zhibin Shi

**Affiliations:** 1 Biomedical Research Center of Xijing University, Xi’an, Shaanxi, China; 2 Department of Orthopedics, The Second Affiliated Hospital, Xi’an Jiaotong University, Xi’an, Shaanxi, China; 3 Department of Neurology, The Second Affiliated Hospital of Xi’an Jiaotong University, Xi’an, Shaanxi, China

**Keywords:** immune biomarkers, inflammatory, memory, neurological diseases, resveratrol

## Abstract

Resveratrol (RES), a naturally occurring polyphenolic compound found in grapes, berries, and peanuts, has attracted considerable interest because of its antioxidant, anti−inflammatory, and neuroprotective properties. This narrative review examines the current evidence regarding the potential effects of RES on memory−related processes and neuroinflammatory biomarkers in major neurological disorders, including Alzheimer’s disease (AD), Parkinson’s disease (PD), multiple sclerosis (MS), and cerebral ischemia. Relevant literature was identified through searches of major scientific databases, and studies addressing the molecular mechanisms, experimental outcomes, and therapeutic implications of RES in these conditions were evaluated. The available evidence indicates that RES can modulate several biological pathways associated with neurodegeneration, including oxidative stress, inflammatory signaling, mitochondrial dysfunction, and neuronal survival. Experimental studies suggest that RES may influence key molecular mediators such as pro−inflammatory cytokines, nitric oxide (NO) signaling, and matrix metalloproteinases, which are implicated in neuronal damage and blood–brain barrier disruption. In preclinical models of AD and PD, RES has been associated with improvements in cognitive performance, reduction of neuroinflammatory markers, and attenuation of neuronal loss. Similarly, studies in MS and cerebral ischemia models indicate that RES may modulate immune responses, reduce oxidative damage, and limit ischemia−related neuronal injury. However, most of the current evidence derives from *in vitro* and animal studies, and clinical data remain limited. Moreover, the low bioavailability of RES and variability in dosing regimens represent important challenges for clinical translation. Therefore, although experimental findings support the potential neuroprotective role of RES, further well−designed clinical studies are required to determine its therapeutic relevance and safety in human neurological disorders. This narrative review was developed through a structured search of PubMed, Scopus, and Web of Science for articles published between 2000 and 2024, focusing on mechanistic, preclinical, and clinical investigations of RES in neurological disorders. This review synthesizes current evidence on the molecular and cellular mechanisms underlying the neuroprotective effects of RES, with particular emphasis on its antioxidant, anti-inflammatory, and immunomodulatory activities. By integrating findings from experimental and clinical research, the review highlights the potential of RES to modulate key pathways involved in neurodegeneration and neuroinflammation. Although further well-designed clinical studies are required to clarify its therapeutic efficacy and translational relevance, the available evidence supports continued investigation of RES as a promising candidate for neuroprotective strategies in neurological disorders.

## Introduction

Neurological ailments, encompassing Alzheimer’s disease (AD), Parkinson’s disease (PD), multiple sclerosis (MS), along with various neurodegenerative and neuroinflammatory conditions, constitute an escalating public health concern globally, primarily attributable to the rising life expectancy and the concomitant increase in age-associated cognitive deterioration. These conditions are distinguished by the progressive degeneration of neurons, neuroinflammatory processes, oxidative stress, mitochondrial dysfunction, and perturbations in immune signaling, culminating in deficits in memory, learning capabilities, and other cognitive functions (1, 2). Notwithstanding considerable advancements in elucidating their molecular pathogenesis, existing therapeutic interventions predominantly address symptomatic relief and are inadequate in effectively arresting or reversing the course of neurodegeneration. As a result, there is a burgeoning interest in naturally occurring bioactive substances endowed with neuroprotective, anti-inflammatory, and antioxidant characteristics that possess the potential to modulate the molecular pathways that underlie neurodegeneration and cognitive dysfunction.

Several polyphenolic compounds, including curcumin, quercetin, and epigallocatechin gallate, have demonstrated neuroprotective, anti-inflammatory, and antioxidant activities that overlap with those reported for resveratrol (RES). These bioactive molecules share the capacity to modulate key signaling pathways involved in oxidative stress, neuroinflammation, and neuronal survival. However, RES has been extensively investigated for its ability to influence interconnected mechanisms such as sirtuin 1 (SIRT1) activation, NF-κB–mediated inflammatory signaling, mitochondrial function, and microglial regulation, processes that collectively link metabolic and inflammatory pathways to neurodegenerative disease progression. In this context, the perspective presented in this review is not that RES acts in isolation among polyphenols, but rather that it serves as a well-characterized model compound through which the interaction between memory-related signaling, neuroinflammation, and immune or neuroimmune biomarkers can be examined in an integrated manner across neurological disorders. RES is derived from Vitis vinifera (grapevine) and Arachis hypogaea (peanut), among other botanical sources such as various berry−producing species. RES, a polyphenolic compound predominantly present in viticultural products, berries, legumes, and red wine, has garnered substantial scholarly interest owing to its multifaceted biological activities, which encompass antioxidant, anti-inflammatory, anti-apoptotic, and immunomodulatory properties (3, 4). Within the central nervous system (CNS), RES has exhibited notable neuroprotective effects across both preclinical and clinical investigations by influencing signaling pathways that are integral to neuronal survival, synaptic plasticity, and cognitive function (5). Mechanistically, RES engages SIRT1, a NAD^+^-dependent deacetylase that governs transcription factors implicated in inflammation, oxidative stress, mitochondrial functionality, and synaptic plasticity, such as PGC-1α, NF-κB, and p53 (6, 7). By facilitating SIRT1 activation, RES bolsters neuronal resilience and fosters synaptic functionality, both of which are essential for memory consolidation and cognitive maintenance (8).

Neuroinflammation represents a fundamental pathological characteristic of numerous neurodegenerative disorders, distinguished by the activation of microglia and astrocytes, alongside the excessive production of pro-inflammatory cytokines, including tumor necrosis factor-alpha (TNF-α), interleukin (IL)-1β, and IL-6 (9). The persistent activation of these glial cells significantly contributes to neuronal damage and synaptic impairment. RES has demonstrated efficacy in attenuating neuroinflammatory responses through the inhibition of NF-κB signaling pathways and the subsequent reduction of cytokine synthesis, thereby safeguarding neuronal integrity (10). Furthermore, RES exerts regulatory effects on immune responses within the CNS, affecting both innate and adaptive immunity by modulating T-cell differentiation and guiding microglial polarization towards neuroprotective phenotypes (11). Such immunomodulatory effects are crucial for reinstating homeostasis in neurodegenerative conditions wherein immune dysregulation assumes a central role. Beyond its anti-inflammatory and immunomodulatory properties, RES significantly influences molecular pathways associated with memory. It facilitates the process of neurogenesis within the hippocampus, augments long-term potentiation (LTP), and elevates the expression of neurotrophic factors including brain-derived neurotrophic factor (BDNF) and NGF, which are crucial for synaptic plasticity and cognitive functions (12). Furthermore, the antioxidant properties of RES alleviate oxidative damage inflicted on neuronal lipids, proteins, and DNA, thus preserving neuronal integrity and functional connectivity (13). Additionally, mitochondrial protection represents a vital mechanism, as RES enhances mitochondrial biogenesis and functionality through the activation of AMP-activated protein kinase (AMPK) and PGC-1α, thereby diminishing the production of reactive oxygen species (ROS) and addressing the energy deficits characteristic of neurodegenerative disorders (14).

Clinical evidence further corroborates the neuroprotective capabilities of RES. Numerous investigations have demonstrated that RES supplementation enhances cognitive function, cerebral perfusion, and neurovascular coupling in both healthy elderly individuals and those exhibiting mild cognitive impairment (15, 16). Moreover, RES generally exhibits a favorable safety profile, and while studies suggest some capacity for crossing the blood–brain barrier (BBB), its clinically significant CNS penetration in humans warrants further investigation. Nevertheless, the limited bioavailability of RES due to expedited metabolic processes constitutes a significant obstacle, thereby necessitating the formulation of nanoencapsulated versions and analogs aimed at optimizing its pharmacokinetic characteristics (17). In aggregate, RES presents itself as a compelling candidate for neuroprotection through its diverse modulation of memory-associated, inflammatory, and immunological biomarkers. By coordinating intricate molecular pathways that involve the activation of SIRT1, the inhibition of NF-κB, and the regulation of neurotrophins, RES induces synergistic effects that mitigate neuronal loss, synaptic impairment, and cognitive deterioration. Elucidating these mechanisms not only enhances our understanding of the molecular foundations of neurodegeneration but also facilitates the development of personalized preventive and therapeutic approaches aimed at addressing neuroinflammatory and neurodegenerative conditions. Although several reviews have examined the effects of RES on cognitive function, neuroinflammation, or immune signaling individually, fewer studies have attempted to integrate these domains within a single framework. The present review therefore aims to provide a consolidated overview of how RES may concurrently influence memory−related outcomes, inflammatory pathways, and immune−associated biomarkers, while situating these findings within the context of existing literature (18), oxidative stress (13), or particular conditions such as AD (12), neglecting to address the interconnected molecular mechanisms that link memory impairment, immune dysregulation, and inflammation in the context of RES’s influence. Furthermore, there exists a notable absence of a systematic review that delineates how RES concurrently affects neurotrophic signaling, cytokine cascades, and the regulation of immune cells across various neurological pathologies. This integrative biomarker-centric approach remains predominantly uncharted. Consequently, the current review seeks to address a significant lacuna by offering a cohesive mechanistic and translational perspective on how RES orchestrates memory-related and immune pathways. It aspires to bridge the gap between preclinical and clinical evidence, emphasizing biomarker-driven methodologies that could inform predictive and preventive strategies in the realms of neurodegenerative and neuroinflammatory diseases. The primary aim of this review is to furnish a thorough and integrative examination of the manner in which RES influences memory-associated, inflammatory, and immune biomarkers to manifest neuroprotective effects in the context of neurological ailments. This investigation aspires to consolidate contemporary molecular and cellular evidence pertaining to the regulatory impacts of RES on memory-related pathways, encompassing neurogenesis, synaptic plasticity, and neurotrophin signaling. Moreover, it endeavors to clarify the anti-inflammatory mechanisms through which RES modulates glial activation and cytokine networks, across both neurodegenerative and neuroinflammatory states. In aggregate, this review establishes a biomarker-centric, mechanistic comprehension of the multifarious actions of RES and underscores its translational potential as a multi-target agent in the prevention and management of neurological disorders. Inter−individual variability plays an important role in shaping RES’s pharmacokinetics, metabolic fate, and downstream biological activity. Emerging evidence indicates that factors such as sex, age, body composition, genetic polymorphisms in metabolizing enzymes, dietary habits, and gut microbiota composition can meaningfully influence systemic exposure to RES and its metabolites (19, 20). Sex−related differences in hormonal regulation, inflammatory tone, and activity of phase II conjugation pathways (e.g., UGTs and SULTs) may contribute to distinct pharmacodynamic responses between males and females. Additionally, lifestyle−related variables, cardiometabolic status, and concomitant medications can further modify absorption, metabolism, and signaling effects. Recognizing these sources of variability is essential for interpreting the heterogeneity observed across preclinical and clinical studies and underscores the need for future biomarker−guided and personalized approaches in evaluating RES’s neuroprotective potential (19, 20).

This article presents a narrative review of the literature examining the neuroprotective effects of RES, with particular attention to its influence on cognitive outcomes, neuroinflammatory pathways, and immune-related biomarkers associated with neurological disorders. This review aims to synthesize current evidence on the neuroprotective potential of RES by examining (1) its modulatory effects on oxidative stress and neuroinflammatory pathways (2), its influence on immune-associated biomarkers relevant to neurological disorders, and (3) its reported effects on cognitive outcomes in preclinical and clinical contexts. A further aim is to integrate these mechanistic and functional domains within a unified framework to contextualize RES’s potential role in neuroprotective research.

## Methods

This article was developed as a narrative review supported by a structured literature search to synthesize current evidence on the neuroprotective effects of RES through modulation of memory-related signaling pathways, neuroinflammation, and immune-associated biomarkers in neurological diseases. The review focused on conditions in which these mechanisms are strongly implicated, including AD, PD, MS, and cerebral ischemia, while also considering mechanistic studies relevant to neurodegeneration and neuroinflammatory processes.

A literature search was conducted using three major scientific databases: PubMed/MEDLINE, Scopus, and Web of Science. The search covered publications from January 2000 through December 2024 and was restricted to articles published in English. Both controlled vocabulary and free-text keywords were used, combined with Boolean operators to refine the search. Core search terms included “resveratrol,” “neuroprotection,” “neurodegeneration,” “neurological disorders,” “cognition,” “memory,” “synaptic plasticity,” “neuroinflammation,” “cytokines,” “NF−κB,” “microglia,” “immune biomarkers,” “autophagy,” and “oxidative stress.” Reference lists of relevant articles and review papers were also examined to identify additional studies not captured during the database search.

Studies were considered eligible if they investigated RES exposure or intervention in the context of neurological disorders or experimental models relevant to neurodegeneration or neuroinflammation and reported outcomes related to cognitive function, synaptic plasticity, inflammatory signaling pathways, oxidative stress, or immune-related biomarkers. Both preclinical (*in vitro* and animal) and clinical studies were included to provide a comprehensive overview of mechanistic and translational evidence. Review articles were consulted primarily for contextual background and to assist in identifying relevant primary studies. Studies were excluded if they did not specifically evaluate RES, focused exclusively on non-neurological conditions without mechanistic relevance to neuroprotection, lacked sufficient methodological detail, or were non–peer-reviewed materials such as editorials or conference abstracts.

Following title and abstract screening, relevant full-text articles were evaluated for inclusion based on their relevance to the thematic domains of the review. Data extracted from eligible studies included study design, experimental model or population, neurological condition investigated, RES dosage and formulation where reported, treatment duration, and primary mechanistic or functional outcomes. The evidence was synthesized qualitatively and organized according to three interconnected domains: memory-related and cognitive outcomes, neuroinflammatory signaling pathways, and immune or neuroimmune biomarkers.

When available, the review also considered factors contributing to inter-individual variability in RES pharmacokinetics and biological responses. These included sex-related differences in metabolism and hormonal regulation, as well as other modifiers such as age, body composition, dietary patterns, gut microbiota composition, baseline inflammatory status, and genetic variability affecting RES metabolism and transport. Such factors were considered important for interpreting translational findings and for guiding future biomarker-informed clinical research.

## RES: chemical structure, pharmacokinetics, and neurobiological relevance

### Chemical characteristics and natural sources of RES

**Figure 1:**
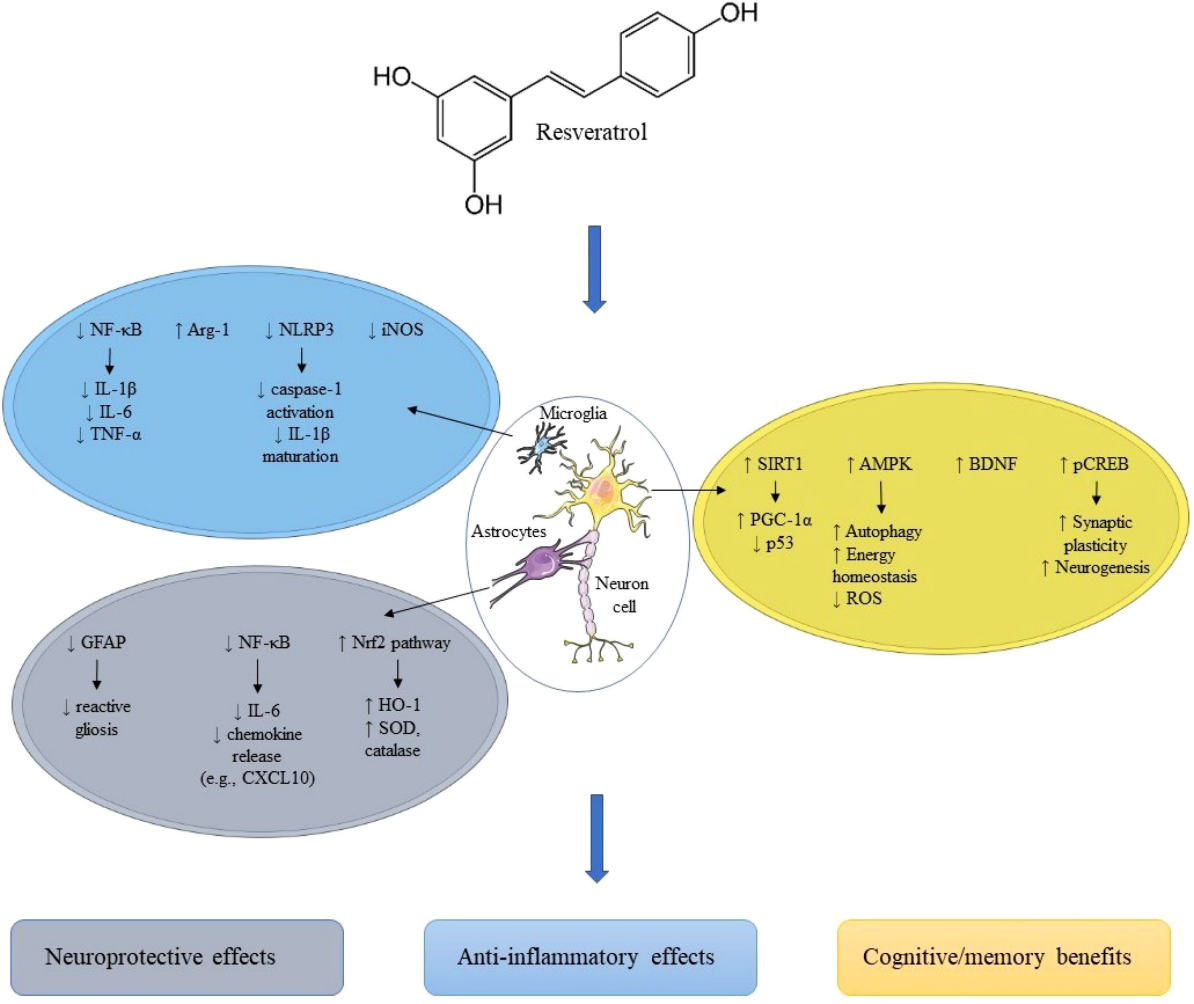
Intracellular mechanisms underlying resveratrol-mediated neuroprotection. Resveratrol activates SIRT1–PGC-1α, AMPK, and BDNF–CREB pathways in neurons, driving mitochondrial biogenesis, antioxidant defense, autophagy, and synaptic plasticity. In microglia, resveratrol inhibits NF-κB and NLRP3 inflammasome signaling, reducing IL-1β, IL-6, and TNF-α and promoting an M1→M2 shift. In astrocytes, resveratrol suppresses GFAP-mediated reactive gliosis and enhances Nrf2-dependent antioxidant responses. These coordinated effects converge to reduce oxidative stress and neuroinflammation and improve neuronal survival and cognition.

RES (3, 5, 4′-trihydroxy-trans-stilbene) constitutes a diminutive stilbenoid polyphenol that is found in nature as both trans and cis isomers, serving as a phytoalexin synthesized by flora in reaction to environmental stressors (21). This compound exhibits lipophilic characteristics while demonstrating limited solubility in water, manifesting in elevated concentrations within grape skins and red wine, and is also detected in various berries, peanuts, and select medicinal flora; its structural attributes (comprising three hydroxyl groups on the stilbene framework) are fundamental to both its antioxidant efficacy and its capacity to engage with an array of protein targets (21, 22) (**Figure 1**). 

### Absorption, metabolism, bioavailability, and blood–brain barrier penetration

Oral RES exhibits significant absorption from the gastrointestinal tract; however, it undergoes extensive first-pass phase II metabolism (including glucuronidation and sulfation) within the intestine and liver, which transforms the parent compound into conjugated metabolites, thereby resulting in markedly low systemic bioavailability of the free aglycone (with bioavailability estimates in humans generally falling estimated at 1-5%) (23, 24). RES and several of its metabolites have been detected in cerebrospinal fluid and brain tissue in diverse animal models, supporting the compound’s capacity to reach CNS compartments under controlled experimental conditions. However, human data remain far more limited and inconsistent, with studies reporting variable or low CNS concentrations that often depend on formulation type, administered dose, and the timing and sensitivity of sampling methods. These discrepancies highlight that, although preclinical models provide strong proof−of−concept for CNS penetration, current clinical findings do not yet confirm reliable or therapeutically significant central exposure. Emerging nanoformulations and bioavailability−enhanced delivery systems show promise in improving absorption and tissue distribution and have been extensively explored in preclinical settings, yet their translational relevance remains under investigation and requires substantiation through well−designed human pharmacokinetic and clinical studies (23, 25).

### Overview of neurobiological effects and preclinical relevance

Mechanistically, RES manifests a multifaceted neuroprotective efficacy that bears significant implications for memory enhancement and the mitigation of neurodegenerative disorders: it activates SIRT1 and AMPK signaling pathways (which enhance mitochondrial biogenesis and metabolic resilience), modulates NF-κB and MAPK signaling cascades (thereby diminishing the production of pro-inflammatory cytokines), augments neurotrophic signaling (BDNF/CREB), and fosters synaptic plasticity along with hippocampal neurogenesis (26, 27). In various rodent models representative of AD, PD, traumatic brain injury, and ischemia, RES has been shown to diminish oxidative damage, mitigate glial activation, reduce the aggregation of pathogenic proteins, and preserve cognitive and behavioral outcomes—findings that collectively bolster its preclinical translational potential (27, 28). However, the inconsistency between the robust outcomes observed in animal studies and the limited, heterogeneous data derived from human trials underscores the necessity for the development of optimized formulations, the validation of CNS biomarkers, and the execution of meticulously designed clinical trials to ascertain the therapeutic viability in patient populations (24).

## The neuroprotective and memory-enhancing effects of RES in AD

Although numerous experimental studies demonstrate the neuroprotective, anti-inflammatory, and antioxidant properties of RES across a variety of neurological disease models, it is important to emphasize that a substantial proportion of this evidence derives from cellular and animal studies. While these investigations provide valuable mechanistic insights, the translation of these findings into consistent therapeutic benefits in humans has been limited and sometimes inconsistent. Differences in bioavailability, metabolism, dosing strategies, and study design may contribute to this discrepancy. Therefore, the following disease-specific sections primarily highlight mechanistic and preclinical evidence, while clinical findings remain comparatively limited and should be interpreted with appropriate caution.

Although numerous studies have reported neuroprotective and memory-enhancing effects of RES in AD models, the current evidence should be interpreted with appropriate caution. Much of the available data is derived from preclinical investigations, including animal and *in vitro* studies, which may not fully replicate the complexity of human AD. In addition, differences in experimental design, dosing regimens, and study populations may contribute to variability in reported outcomes. Therefore, while the existing findings suggest promising therapeutic potential, further well-designed clinical studies are required to confirm the efficacy and translational relevance of RES in improving cognitive function and neuroprotection in patients with AD.

The pathological aggregation of amyloid-β (Aβ) peptides within the cerebral environment fosters a state of chronic neuroinflammation, predominantly orchestrated by activated astrocytes and microglia, which consequently undermines neuronal communication and the integrity of synaptic structures. In a rat model of AD induced by Aβ25–35, the administration of RES markedly attenuated neuroinflammation and reinstated neural homeostasis. This compound diminished the activation levels of astrocytes and microglia, augmented the quantity of Doublecortin-positive cells (an indicator of heightened hippocampal neurogenesis) and facilitated improved performance in behavioral assessments related to spatial navigation and memory acquisition. Moreover, the observed upregulation of SIRT1 and choline acetyltransferase (ChAT) expression implied that RES fosters both neurogenesis and the enhancement of cholinergic functionality, thereby contributing to the amelioration of cognitive outcomes. These results underscore the dual role of RES as both an anti-inflammatory agent and a neurogenic promoter, crucially preserving the integrity and functionality of the hippocampus (29) (**Table 1**). Building upon these mechanisms, investigations concerning dihydro-RES (DHR), a polyphenolic metabolite derived from RES, have further clarified its influence on neuroinflammatory pathways in AD. Employing lipopolysaccharide (LPS)- and AD-induced murine models, DHR exhibited a significant attenuation of NLRP3 inflammasome activation, a principal contributor to neuroinflammation and pyroptotic cell demise. DHR promoted autophagy and mitophagy processes through Bnip3-dependent signaling and the phosphorylation of ULK, thereby enhancing mitochondrial quality control and mitigating oxidative stress. Behavioral evaluations substantiated that DHR significantly enhanced cognitive functions such as learning and memory, while biochemical investigations indicated a reduction in amyloid precursor protein (APP) pathology and the synthesis of inflammatory cytokines. The inhibition of mitophagy or the activation of NLRP3 negated DHR’s therapeutic benefits, thereby substantiating that its neuroprotective effects are mediated through the restoration of autophagy-mitophagy and the suppression of inflammasome activity. This body of evidence positions DHR as a formidable modulator of innate immune signaling and mitochondrial equilibrium in the context of AD (30). It is important to distinguish between native RES and structurally related compounds, derivatives, or formulation−based modifications that are sometimes investigated alongside it. Several studies have explored RES analogs, synthetic derivatives, or advanced delivery systems such as nanoformulations in an effort to improve stability, bioavailability, and tissue distribution. While these approaches may enhance pharmacokinetic properties or biological activity, such compounds should not be considered pharmacologically identical to RES. Structural modifications or formulation strategies may alter pharmacodynamic effects, metabolic pathways, and safety profiles. Therefore, throughout this review, findings derived from RES analogs, derivatives, or specialized formulations are interpreted as related but distinct lines of investigation rather than direct evidence of the effects of native RES itself.

**Table 1:** Summary of studies investigating the neuroprotective and memory-enhancing effects of resveratrol in Alzheimer’s disease models.

Study/model	Intervention (dose & duration)	Mechanisms & biomarkers	Cognitive/behavioral outcomes	Key findings/implications	Reference
Aβ25–35-induced rat model of AD	Resveratrol 30 mg/kg, 4 weeks	↑ SIRT1, ↑ Doublecortin (neurogenesis), ↓ reactive astrocytes & microglia, ↑ ChAT (choline acetyltransferase)	Improved spatial and learning memory (Morris water maze, radial arm maze)	Resveratrol reduces neuroinflammation, restores hippocampal homeostasis, enhances neurogenesis, and improves memory through SIRT1-dependent mechanisms.	(29)
LPS- and APP/PS1-induced AD mice; primary microglia	Dihydro-resveratrol (dose not specified)	↓ NLRP3 inflammasome, ↑ Bnip3-dependent mitophagy, ↑ ULK phosphorylation	Restored spatial learning and memory (Morris water maze, open-field test)	Dihydro-resveratrol suppresses neuroinflammation and amyloid pathology via activation of mitophagy; a promising anti-neuroinflammatory therapeutic for AD.	(30)
APP/PS1 transgenic mice	ApoE-modified liposomes co-delivering resveratrol and salidroside	↑ Brain uptake, ↓ oxidative and inflammatory markers, ↑ anti-apoptotic factors	Improved learning and memory performance	ApoE-Res/Sal-Lips improve BBB permeability and therapeutic efficacy; a novel nanodelivery platform for AD treatment.	(31)
AD rat model	Intranasal *in situ* gelling liquid crystal formulation of resveratrol	↓ ILs (inflammatory cytokines), ↓ amyloid formation	Improved learning and memory after intranasal administration	Intranasal delivery enhances bioavailability and ameliorates neuroinflammation and memory loss via non-invasive administration route.	(32)
Theoretical model of hippocampal neurogenesis	Resveratrol	↑ SIRT1 → ↑ Wnt signaling → ↑ neural stem cell activation & hippocampal neurogenesis	Predicted improvement in cognitive performance	Resveratrol activates SIRT1–Wnt synergy to enhance hippocampal neuroregeneration and memory restoration in AD.	(33)
AlCl_3_-induced AD mouse model with Environmental Enrichment (EE)	Resveratrol 200 mg/kg ± EE, 8 weeks	↓ Aβ, ↑ Nrf2 (antioxidant defense), modulation of neurochemical	Improved contextual fear memory; enhanced performance in resveratrol ± EE groups	Resveratrol and EE synergistically improve memory and reduce amyloid burden via antioxidative and neurochemical regulation.	(34)
Streptozotocin-induced AD model	Resveratrol-loaded bilosomes (optimized oral formulation)	↓ COX-2, ↓ IL-6, ↓ Aβ, ↓ Tau, ↓ GFAP & microglial count	Enhanced memory (Y-maze, Morris water maze)	Oral resveratrol-bilosomes improve bioavailability and exert anti-inflammatory, anti-amyloid, and cognitive-protective effects in AD.	(35)
AlCl_3_ + D-galactose-induced AD mouse model	Melatonin (80 mg/kg), Resveratrol (40 mg/kg), alone or combined	↑ AChE, ↑ BDNF, ↑ CREB signaling	Improved recognition memory (NORT) and passive avoidance (PAT)	Combined melatonin + resveratrol treatment produced additive benefits on recognition memory via cholinergic and BDNF-CREB pathways.	(36)
High-fat diet (HFD)-induced AD in WT and 5XFAD mice	Resveratrol 0.1% in diet, 16 weeks	↓ amyloidogenic APP processing, ↓ Tau pathology, normalization of proteasomal activity	Prevented memory loss (Morris water maze)	Resveratrol counters HFD-induced amyloid and Tau pathology; protects cognition by restoring proteolytic balance.	(37)
APP/PS1 transgenic mice	RVG/TPP-modified RBC membrane-coated nanocarriers loaded with resveratrol	Targeted mitochondrial delivery, ↓ ROS, ↓ Aβ mitochondrial stress	Improved memory after systemic administration	Mitochondria-targeted nanodelivery enables BBB crossing, reduces oxidative stress, and restores memory in AD mice.	(38)
Rat model of combined diabetes and AD (STZ + Aβ1–40)	Resveratrol (dose not specified) ± SIRT1 inhibitor (EX527)	↑ SIRT1, ↓ IL-1β, ↓ IL-6, ↓ MDA, ↑ SOD, ↑ GSH, ↑ ChAT	Inhibited memory impairment	Resveratrol protects against diabetes-induced AD via SIRT1 activation and oxidative/inflammatory suppression.	(39)
Tg6799 (5XFAD) transgenic mice	Resveratrol 60 mg/kg, 60 days	↓ Aβ42, ↓ β-secretase 1, ↓ APP cleavage products	Improved spatial working and reference memory (Y-maze, Morris water maze)	Resveratrol reduces amyloid plaque formation and improves cognition in familial AD model.	(40)
Angiotensin II-induced early AD rat model	Resveratrol 10 mg/kg/day, 2 weeks	↓ ROS, ↑ BDNF, ↓ GSK-3β^Y216, ↓ caspase-3, ↓ Aβ precursors	Restored hippocampal-dependent contextual memory	Resveratrol reduces oxidative stress, modulates NOX2/SOD2 balance, and rescues early AD-related memory loss.	(41)
AD mouse model + hUC-MSC transplantation	Resveratrol combined with hUC-MSCs	↑ SIRT1, ↑ PCNA, ↓ ac-p53, ↓ p21/p16, ↓ apoptosis, ↑ neurogenesis	Improved spatial learning and memory (Morris water maze)	Resveratrol enhances stem cell engraftment, hippocampal neurogenesis, and neural repair via SIRT1 activation.	(42)
Intrahippocampal Ibotenic Acid-Induced Alzheimer’s Model (Male Wistar Rats)	Resveratrol (20 mg/kg, i.p., duration not specified)	↓ Oxidative stress markers (nitrite, PCO, MDA); ↑ antioxidant enzymes; normalization of NR2A/NR2B, α7-nAChR, and m1AChR expression	Improved spatial learning and memory (behavioral tests)	Resveratrol mitigated IBO-induced cholinergic and glutamatergic dysfunction, reduced oxidative stress, and preserved hippocampal neurons, suggesting neuroprotection in AD.	(43)
β-Amyloid (Aβ_1-42_)-Induced Alzheimer’s Mouse Model	Resveratrol (dose not specified; Aβ microinfusion in CA1 region)	↓ PDE4A/B/D expression; ↑ cAMP, PKA, pCREB, BDNF, Bcl-2; ↓ neuroinflammation and apoptosis	Improved learning and memory performance	Resveratrol ameliorated Aβ-induced cognitive deficits via PDE4–cAMP–PKA–CREB–BDNF pathway modulation, promoting neuronal survival and plasticity.	(44)
Lipopolysaccharide (LPS)-Induced Alzheimer’s Disease Mouse Model	Resveratrol (4 mg/kg, i.p., 7 days)	↑ Estradiol levels; ↑ Neprilysin (NEP) expression (Aβ degradation enzyme)	Improved working, spatial, and locomotor memory (Y-maze, object recognition, open field)	Resveratrol enhanced estradiol and NEP expression, promoting Aβ clearance and reversing LPS-induced cognitive impairment.	(45)
AβPP/PS1 Transgenic Mouse Model of Familial Alzheimer’s Disease	Resveratrol (oral administration; long-term)	↑ SIRT1 and AMPK activation; ↑ mitochondrial complex IV; mild ↑ IL-1β and TNF; unchanged oxidative stress markers	Prevented memory decline (object recognition test)	Resveratrol reduced amyloid burden and preserved cognition via SIRT1/AMPK-mediated mitochondrial and neuroprotective mechanisms.	(46)

Aβ, Amyloid beta; AβPP, Amyloid beta precursor protein; AβPP/PS1, Amyloid beta precursor protein/presenilin-1 transgenic mice; Aβ_1-42_, Amyloid beta peptide 1–42; AChE, Acetylcholinesterase; AD, Alzheimer’s disease; AMPK, AMP-activated protein kinase; Ang-II, Angiotensin II; APP/PS1, Amyloid precursor protein/presenilin-1 transgenic model; AT8, Antibody detecting phosphorylated tau, BDNF; Brain-derived neurotrophic factor; Bcl-2, B-cell lymphoma 2 (anti-apoptotic protein); Bnip3, BCL2 interacting protein 3 (mitophagy marker); CA1, Cornu Ammonis region 1 of hippocampus; ChAT, Choline acetyltransferase; COX-2 – Cyclooxygenase-2; CREB, cAMP response element-binding protein; DCX, Doublecortin (marker of neurogenesis); ELISA, Enzyme-linked immunosorbent assay; GSK-3β, Glycogen synthase kinase 3 beta; HFD, High-fat diet; hUC-MSCs, Human umbilical cord mesenchymal stem cells; IBO, Ibotenic acid; IL-1β, Interleukin-1 beta; IL-6, Interleukin-6; LPS, Lipopolysaccharide; MDA, Malondialdehyde; MPTP – 1-Methyl-4-phenyl-1,2,3,6-tetrahydropyridine, NEP, Neprilysin; NF-κB, Nuclear factor kappa-light-chain-enhancer of activated B cells; NMDA, N-Methyl-D-aspartate receptor; NR2A/NR2B, NMDA receptor subunits 2A and 2B; NLRP3, NOD-, LRR-, and pyrin domain-containing protein 3 inflammasome; pCREB, Phosphorylated cAMP response element-binding protein; PDE4, Phosphodiesterase type 4; PKA, Protein kinase A; PKG, Protein kinase G; ROS, Reactive oxygen species; RSV, Resveratrol; SIRT1, Sirtuin 1 (NAD^+^-dependent deacetylase); STZ, Streptozotocin; TNF, Tumor necrosis factor; TNF-α, Tumor necrosis factor alpha; TPP, Triphenylphosphonium (mitochondrial-targeting ligand); α7-nAChR, Alpha7 nicotinic acetylcholine receptor; m1AChR, Muscarinic acetylcholine receptor M1 subtype; p-Tau, Phosphorylated tau protein; BDL, Bilosome drug-loaded formulation; PDE4A/B/D, Phosphodiesterase-4 subtypes A, B, and D; SOD, Superoxide dismutase; CAT, Catalase; GSH, Glutathione; MPO, Myeloperoxidase; ApoE, Apolipoprotein E; EE, Environmental enrichment; H89, PKA inhibitor; KT5823, PKG inhibitor; MRC IV, Mitochondrial respiratory chain complex IV; Tg6799/5XFAD, Transgenic mouse model of familial Alzheimer’s disease; NE, Norepinephrine; MAO-B, Monoamine oxidase B. ↓, Decrease; ↑, Increase.

To address the pharmacokinetic constraints associated with RES and to augment its delivery to the CNS, innovative nanotechnology-based methodologies have been developed. In a particular investigation, liposomal formulations co-encapsulating both RES and salidroside underwent surface modification with apolipoprotein E (ApoE) to enhance specificity, solubility, and the permeability of the BBB. *In vitro* assays revealed a significant increase in the cellular uptake of these nanoformulations by neuronal and endothelial cell types, whereas *in vivo* studies conducted on APP/PS1 mouse models demonstrated enhanced BBB transport and targeted accumulation within the brain. The ApoE-modified liposomes mitigated oxidative stress, neuroinflammation, and apoptosis, thereby resulting in a reduction of cognitive decline and an improvement in memory performance. This approach highlights the critical importance of sophisticated delivery systems in augmenting the therapeutic efficacy of polyphenols in the context of AD management (31). In addition to nanocarrier methodologies, intranasal administration has emerged as a compelling strategy to circumvent systemic metabolism while enabling direct targeting of cerebral tissue. A novel *in situ* gelling liquid-crystal formulation of RES, derived from oleic acid and surfactant systems, demonstrated advantageous biophysical and structural characteristics, as confirmed through polarized light microscopy, small-angle X-ray scattering, and transmission electron microscopy (TEM) analyses. Following intranasal delivery, this formulation markedly enhanced cognitive functions, including learning and memory, in animal models of AD and mitigated neuroinflammation by downregulating pro-inflammatory cytokines such as IL. This methodology offers a non-invasive, efficient, and patient-centric delivery mechanism that optimizes RES bioavailability and therapeutic efficacy in the context of neurodegenerative disorders (32).

At the molecular level, the neuroregenerative properties of RES are intricately associated with its ability to activate SIRT1 and modulate Wnt signaling pathways—critical determinants of neural stem cell proliferation and differentiation. The aberrant regulation of Wnt signaling is a defining characteristic of AD, contributing to the disruption of hippocampal neurogenesis and subsequent cognitive deterioration. By enhancing the activity of SIRT1, RES facilitates Wnt-mediated transcriptional activation of neurogenic genes, thereby promoting the proliferation of neural progenitor cells and reinstating hippocampal plasticity. This synergistic interplay between SIRT1 and Wnt signaling not only aids in neuroprotection but also in regeneration, providing a mechanistic framework through which RES may ameliorate memory deficits and the structural brain damage observed in AD (33). In experimental neuroscience, environmental enrichment (EE) refers to housing conditions designed to increase sensory, cognitive, and physical stimulation compared with standard laboratory environments. Such paradigms typically include larger cages, social interaction, running wheels, tunnels, toys, and periodically changing objects that encourage exploration and physical activity. EE has been widely used to enhance neuroplasticity, promote cognitive function, and modulate neuroinflammatory and oxidative stress pathways in animal models. In the present context, aluminum chloride (AlCl_3_) was employed to induce neurotoxicity and cognitive impairment in rodents, a commonly used experimental approach to model certain pathological features associated with neurodegenerative processes, including oxidative stress, neuroinflammation, and memory deficits. Recent investigations persist in substantiating the therapeutic significance of RES in mitigating cognitive deterioration and neuropathological processes associated with AD. Within a model of AD induced by AlCl_3_, the efficacy of RES was assessed in conjunction with EE to ascertain their respective independent and synergistic impacts on cognitive function and neurochemical profiles. Although exposure to AlCl_3_ resulted in significant deficits in contextual fear memory, supplementation with RES significantly enhanced memory and learning capabilities. Importantly, the combined intervention of EE and RES led to an increase in Nrf2 and Aβ protein expression, indicating an improved antioxidant response and a potential alteration in amyloid metabolism. Intriguingly, solitary interventions with either RES or EE demonstrated greater efficacy in enhancing memory compared to their combined application, suggesting the possibility of competitive or threshold interactions in the modulation of neuroplasticity. These findings underscore the potential of RES to rehabilitate hippocampal functionality and cognitive integrity through mechanisms of oxidative equilibrium and synaptic facilitation (34).

Further augmentation of RES bioavailability has been investigated through bilosome-mediated oral delivery mechanisms. Within a streptozotocin-induced AD model, RES-encapsulated bilosomes exhibited enhanced pharmacological effectiveness in comparison to traditional suspensions. The optimized formulation attained superior physicochemical characteristics, elevated drug entrapment efficiency, and enhanced stability. *In vivo* behavioral assessments, encompassing Y-maze and Morris water maze evaluations, indicated a noteworthy restoration of cognitive and learning abilities. Biochemically, administration of RES-encapsulated bilosomes resulted in diminished levels of COX2, IL-6, Aβ, and Tau protein, in conjunction with reduced activation of astrocytes and microglia. These findings imply that the nano-encapsulation of RES through bilosomes significantly improves its systemic absorption, penetration across the BBB, and overall neuroprotective efficacy (35). The synergistic potential of RES and melatonin has been examined in a murine model of AD induced by AlCl_3_ and D-galactose. While both compounds independently enhanced cognitive performance, their concurrent administration yielded markedly superior outcomes in recognition memory and passive avoidance behavior. Mechanistically, this observed synergism was correlated with augmented cholinergic activity, upregulation of BDNF, and activation of CREB signaling within the prefrontal cortex. These molecular alterations imply that RES and melatonin collaboratively bolster neurotrophic and synaptic signaling pathways, thereby facilitating memory consolidation and synaptic resilience (36).

Beyond the implications of genetic and amyloid models, dietary-induced metabolic stress is increasingly acknowledged as a significant contributor to the pathogenesis of AD. In a murine model subjected to a high-fat diet (HFD), the administration of RES demonstrated a marked efficacy in mitigating diet-induced neurodegeneration and cognitive deficits. This bioactive compound was observed to avert memory deterioration in both wild-type and transgenic 5XFAD mice, while concurrently reducing amyloid and tau pathologies and modulating proteolytic mechanisms, specifically the ubiquitin–proteasome pathway. The capacity of RES to restore normal proteolytic activity under HFD conditions indicates its bifunctional role in preserving proteostasis and alleviating neurodegeneration associated with metabolic stress. These results broaden the therapeutic potential of RES to address cognitive decline linked to obesity and the metabolic factors contributing to the progression of AD (37). To tackle the enduring challenge of effective targeting of brain and mitochondrial structures, a groundbreaking dual-modified biomimetic nanosystem has been recently formulated for the delivery of RES. This innovative system, which consists of NLCs that are coated with red blood cell membranes and functionally enhanced with rabies virus glycoprotein (RVG29) and triphenylphosphine (TPP), exhibited precise targeting capabilities towards neuronal mitochondria following systemic administration. These multifunctional nanoparticles demonstrated a remarkable ability to traverse the BBB, effectively localizing within neuronal mitochondria while ensuring a sustained release of antioxidants. The encapsulation of RES within this nanosystem significantly mitigated Aβ-induced mitochondrial oxidative stress, restored mitochondrial functionality, and enhanced cognitive performance in APP/PS1 murine models. This methodological approach signifies a transformative advancement in AD nanotherapy by amalgamating biomimetic targeting with organelle-specific drug delivery to surmount systemic obstacles and attain neuroprotection at the mitochondrial level (38).

An increasing body of evidence consistently reinforces the complex neuroprotective mechanisms of RES across various experimental paradigms of AD, particularly in contexts complicated by concomitant metabolic or vascular stressors. In a rat model that integrates diabetes mellitus (DM) and AD, RES demonstrated significant protective effects via the activation of Sirt1, a pivotal regulator of cellular resilience against stress and longevity. The intervention markedly reinstated Sirt1 expression, diminished acetylcholinesterase (AChE) activity, lipid peroxidation (MDA), and pro-inflammatory cytokines (IL-1β, IL-6), while concurrently enhancing antioxidant enzymes like superoxide dismutase (SOD), glutathione (GSH), and ChAT. Notably, pharmacological inhibition of Sirt1 (EX527) diminished these protective effects, thereby corroborating that Sirt1-mediated signaling represents a fundamental molecular pathway for RES’s neuroprotection in the milieu of diabetes-related cognitive deterioration. This indicates that RES may function as a molecular conduit linking metabolic regulation and neurodegeneration, addressing shared pathogenic mechanisms such as oxidative stress and inflammation (39). In transgenic Tg6799 (5xFAD) murine models, the administration of RES (60 mg/kg over a 60-day period) resulted in a significant decrease in the amyloid plaque burden, a reduction in levels of Aβ42 and β-secretase (BACE1) expression, as well as a downregulation of APP and its associated cleavage products. Behavioral evaluations demonstrated a notable improvement in both spatial working memory and reference memory as assessed through Y-maze and Morris water maze paradigms, thereby indicating a functional restoration of hippocampal integrity. Notably, the effects of RES were confined to cognitive functions, with no discernible impact on motor performance. These findings substantiate its potential as an anti-amyloidogenic and cognition-preserving agent, capable of modulating the amyloidogenic pathway and mitigating Aβ-induced neuronal toxicity (40).

The vascular and oxidative stress elements associated with AD were subsequently scrutinized within the framework of an angiotensin II (Ang-II)-induced early-stage AD model. In this regard, RES demonstrated effects analogous to those of the antihypertensive medication losartan, as it diminished the production of ROS within the nucleus tractus solitarius, augmented the expression of BDNF in the hippocampus, and reinstated contextual memory capabilities. Molecular evaluations uncovered a reduction in levels of APP, active caspase-3, and GSK-3β (Y216), coupled with an elevation in Akt (S473) phosphorylation, implicating the inhibition of apoptotic and pro-amyloidogenic signaling pathways. Both RES and losartan were found to mitigate Tau (T231) hyperphosphorylation and to facilitate redox homeostasis through the suppression of NOX2 and the upregulation of superoxide dismutase 2 (SOD2). These results elucidate that RES not only alleviates oxidative injury but also serves as a vascular-neuroprotective agent, potentially offering advantages for hypertensive patients predisposed to AD (41). Another promising research direction pertains to stem cell-mediated neurorepair enhanced by RES. In a transplantation model utilizing human umbilical cord-derived mesenchymal stem cells (hUC-MSC) for AD, RES significantly augmented cell engraftment, neuronal viability, and hippocampal neurogenesis. The co-administration of treatments resulted in marked improvements in cognitive performance as measured by the Morris water maze, which paralleled the upregulation of SIRT1 and PCNA, alongside the downregulation of p53, acetyl-p53, p21, and p16. These alterations denote a coordinated attenuation of cellular senescence and apoptosis, in conjunction with the activation of proliferative and regenerative pathways. Therefore, RES seems to facilitate a neurobiological milieu conducive to stem cell incorporation and functionality, presenting a synergistic therapeutic framework that amalgamates neuroprotection with neural regeneration (42).

In a complementary model of cholinergic dysfunction and excitotoxicity induced by ibotenic acid (IBO) in rats, the administration of RES (20 mg/kg) significantly mitigated impairments in learning and memory, reinstated open-field activity, and diminished oxidative stress biomarkers such as nitrite, protein carbonyl, and MDA. Molecular analyses indicated a restoration of NMDA receptor subunit levels (NR2A/NR2B) and cholinergic receptor expression, notably α7-nAChR and m1AChR, which are critical mediators of synaptic plasticity. Histopathological assessments demonstrated that RES effectively inhibited hippocampal neuronal loss and the thinning of the pyramidal layer, thereby corroborating its efficacy in preserving both structural and functional neuronal integrity. These findings highlight the dual capacity of RES to counteract glutamatergic excitotoxicity and restore cholinergic signaling, both of which are integral to the pathophysiology of AD (43). In Aβ_1-42_-induced AD murine models, the administration of RES effectively reinstated cognitive and mnemonic functions that were compromised due to hippocampal Aβ microinfusion, thereby elucidating a crucial role of the cAMP–CREB–BDNF signaling pathway in its underlying mechanism of action. More specifically, exposure to Aβ markedly augmented the expression of phosphodiesterase-4 (PDE4A/B/D), diminished protein kinase A (PKA) levels, and heightened indicators of neuroinflammation and apoptotic processes. The treatment with RES mitigated these alterations by inhibiting the various subtypes of PDE4, which resulted in an elevation of intracellular cAMP, pCREB, and BDNF levels, in conjunction with an increase in Bcl-2 expression, indicating a robust anti-apoptotic effect. The obliteration of the beneficial effects of RES through the application of the PKA inhibitor (H89) and not the PKG inhibitor (KT5823) corroborated that its effects were specifically facilitated through the PDE4–cAMP–PKA–CREB–BDNF signaling axis. These findings imply that RES promotes neuronal viability and cognitive function by thwarting the degradation of cAMP, thereby fostering LTP and neuroplasticity, which are essential for the preservation of memory in AD (44).

**Figure 2:**
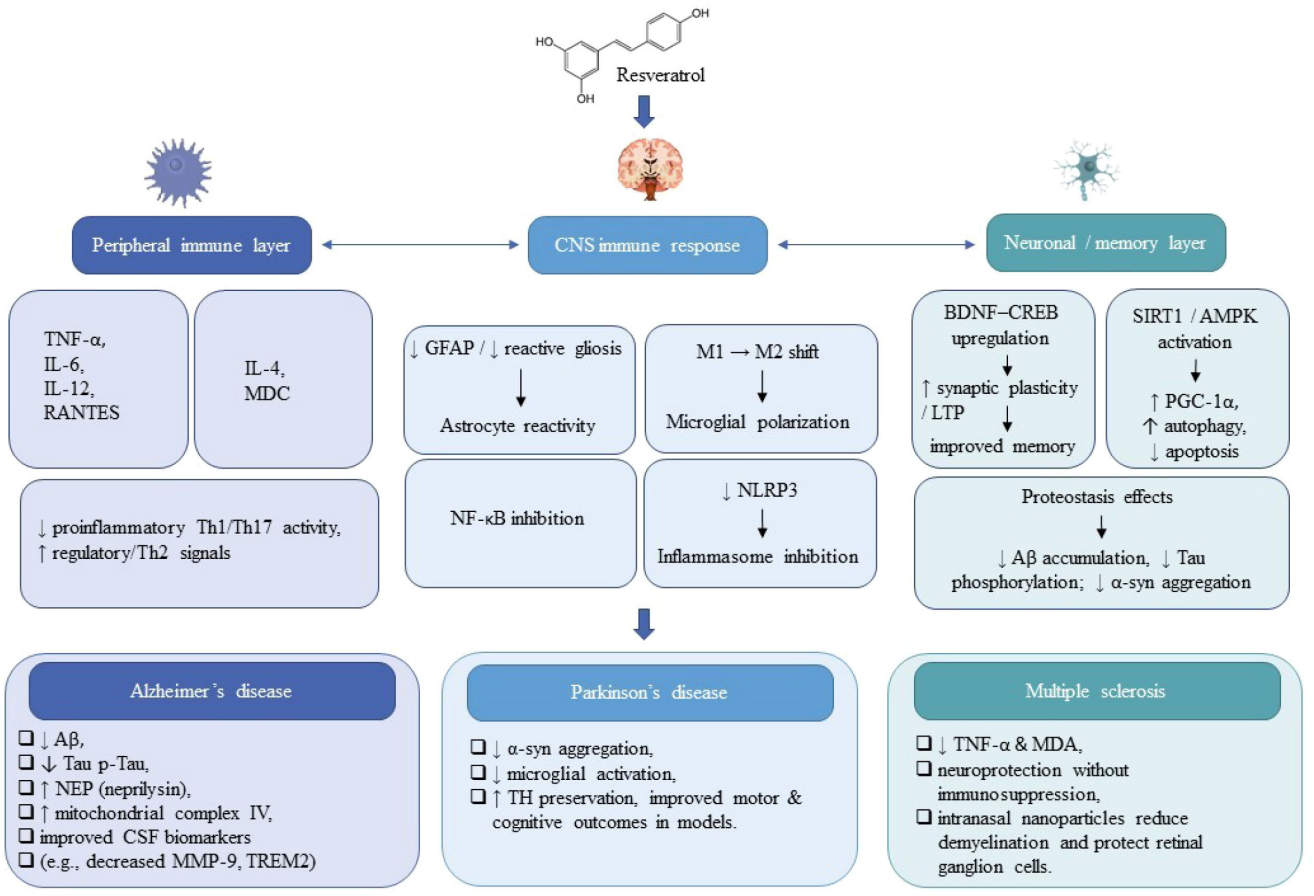
Systems-level model of resveratrol’s modulation of the inflammation–immunity–memory axis across neurodegenerative diseases. Resveratrol reshapes peripheral immunity (↓ TNF-α, IL-6; ↑ IL-4, MDC), attenuates CNS innate immune activation via NF-κB and NLRP3 inhibition, and promotes microglial M2 polarization. Neuronal effects include activation of SIRT1/AMPK and BDNF–CREB signaling, leading to improved synaptic plasticity and mitochondrial function. Disease-specific benefits include reduced Aβ and tau pathology in Alzheimer’s disease, decreased α-synuclein toxicity in Parkinson’s disease, and antioxidant protection in multiple sclerosis. ↓, Decrease; ↑, Increase.

In a LPS-induced inflammatory model of AD, RES demonstrated a dual regulatory effect on hormonal and enzymatic pathways by targeting estradiol and neprilysin (NEP) pathways. Exposure to LPS resulted in the impairment of various cognitive domains (working memory, nonspatial memory, and locomotor activity), whereas administration of RES (4 mg/kg for a duration of 7 days) ameliorated these behavioral deficits as evidenced by performance in Y-maze, object recognition, and open field assessments. On a biochemical level, RES significantly augmented levels of estradiol and NEP—two interdependent components that are essential for the degradation of amyloid-beta (Aβ). Given that estrogen signaling is recognized for its capacity to enhance NEP expression, the phytoestrogenic properties of RES likely facilitated an increase in NEP-mediated Aβ clearance, thereby diminishing amyloid accumulation and alleviating cognitive decline. These findings underscore the potential of RES to act as a phytoestrogenic modulator, integrating hormonal regulation and amyloid metabolism to confer neuroprotection in the context of inflammation-associated AD (45). In alignment with these findings, investigations utilizing the AβPP/PS1 transgenic murine model exhibited that prolonged oral administration of RES significantly mitigated memory deficits and diminished amyloid accumulation. This intervention also resulted in an upregulation of mitochondrial complex IV expression, indicative of enhanced mitochondrial bioenergetic function. Molecular investigations elucidated that these neuroprotective outcomes were primarily facilitated through the activation of SIRT1 and AMPK signaling pathways, both of which serve as pivotal regulators of cellular metabolism, autophagy, and neuronal viability. Notably, RES elicited modest elevations in IL-1β and TNF expression, implying a finely calibrated modulation of inflammatory pathways rather than a complete inhibition—potentially signifying adaptive immune responses that foster tissue repair. No noteworthy changes were detected in oxidative stress biomarkers, thereby suggesting that the principal effects of RES were accomplished through metabolic reprogramming and mitochondrial stabilization, rather than through direct antioxidant mechanisms (46). The cumulative preclinical data substantiates the characterization of RES as a pleiotropic compound that concurrently targets multiple mechanisms pertinent to AD, including neuroinflammation, compromised neurogenesis and synaptic functionality, mitochondrial dysfunction, and proteostatic disruption, which likely contributes to its consistently observable memory-preserving effects across various experimental frameworks. Nevertheless, the progression to clinical application is hampered by variability in dosage and pharmacokinetic data, a scarcity of long-term safety information at therapeutic levels, discrepancies in the relevance of animal models to sporadic human AD, and a dearth of rigorous clinical investigations that demonstrate cognitive advantages. Upcoming research endeavors should emphasize the necessity for standardized dose-response investigations, comparative analyses of delivery methodologies, assessments in aged and comorbid models that more accurately simulate human AD, the identification of mechanistic biomarkers (including CSF/plasma SIRT1, mitophagy and proteostasis indicators, as well as neuroinflammatory profiles), and meticulously crafted early-phase human trials that integrate optimized formulations alongside measures of target engagement. In conclusion, the aggregation of preclinical investigations concerning these studies indicates that RES functions as a robust multi-target candidate for the preservation of memory in AD; however, progression towards clinical significance necessitates the resolution of bioavailability challenges, the standardization of experimental methodologies, and the development of translational biomarkers alongside safety evidence (**Figure 2**). Preclinical studies provide substantial evidence that RES may modulate several pathological mechanisms implicated in AD, including oxidative stress, neuroinflammation, amyloid−β accumulation, and synaptic dysfunction. However, despite these promising findings in experimental models, clinical trials evaluating RES in patients with AD have yielded variable outcomes, often limited by small sample sizes, short treatment durations, and issues related to bioavailability. Consequently, further well−designed clinical studies are required to determine whether the neuroprotective effects observed in preclinical models can translate into meaningful therapeutic benefits in human populations.

## The effects of RES on inflammation–immunity axis in AD

RES has been recognized as a significant modulator of neuroinflammatory and immune mechanisms in AD, functioning predominantly through the activation of SIRT1 and its subsequent molecular targets.

Clinical studies. In a clinical trial spanning 52 weeks, which involved individuals diagnosed with mild to moderate AD, the administration of RES (up to 1 g administered bi-daily) markedly mitigated the decline in levels of CSF Aβ42 and cognitive performance, concurrently influencing various inflammatory mediators. In particular, RES resulted in a reduction of CSF matrix metalloproteinase-9 (MMP-9) levels while enhancing the concentrations of macrophage-derived chemokine (MDC), IL-4, and fibroblast growth factor-2 (FGF-2), which signifies a transition towards adaptive immunity and anti-inflammatory signaling pathways. These observations imply that the activation of SIRT1 may not solely diminish neuroinflammation but also facilitate the maintenance of neuroimmune homeostasis in the context of AD (47) (**Table 2**). Complementary evidence derived from an additional multicenter, placebo-controlled clinical trial further substantiates the anti-inflammatory and neuroprotective properties of RES. In this investigation, RES was found to diminish CSF concentrations of TREM2, a biomarker indicative of microglial activation, and MMP-9, while concurrently reducing markers of neuronal damage including neuron-specific enolase and phosphorylated neurofilaments. Furthermore, the elevation in angiogenin levels and the reduction in cathepsin D suggest enhancements in vascular integrity and autophagic equilibrium. Collectively, these alterations in biomarkers bolster the argument for RES’s efficacy in attenuating microglial hyperactivation, neuronal impairment, and inflammatory processes in patients diagnosed with AD (48).

**Table 2:** Summary of studies on resveratrol and inflammation–immunity axis in Alzheimer’s disease.

Study/Model	Intervention (dose & duration)	Mechanisms & biomarkers	Key findings/Implications	Reference
Human clinical trial (mild–moderate AD patients)	Resveratrol up to 1 g twice daily for 52 weeks	↓ CSF MMP-9; ↑ IL-4, MDC, FGF-2; ↓ plasma IL-12p40, IL-12p70, RANTES; SIRT1 activation; modulation of adaptive immunity	Resveratrol reduced neuroinflammation and promoted adaptive immune signaling via SIRT1 activation, suggesting anti-inflammatory and neuroprotective potential.	(47)
Human clinical trial (subset of phase II AD study)	Resveratrol for 52 weeks (n = 30) vs placebo (n = 21)	↓ CSF TREM2, ↓ neuron-specific enolase, ↓ phospho-neurofilament, ↓ MMP-9, ↓ Cathepsin D, ↑ Angiogenin	Resveratrol decreased markers of neurodegeneration, inflammation, and microglial activation, supporting its anti-inflammatory and neuroprotective role in AD.	(48)
Rat AD model (AlCl_3_-induced)	Resveratrol–selenium nanoparticles (RSV-SeNPs) for 60 days	↑ SIRT1; ↓ miR-134; ↓ STAT3 and IL-1β; ↓ GSK3β activity; ↑ PI3K signaling; improved mitochondrial function	RSV-SeNPs alleviated neuroinflammation and neurotoxicity via SIRT1/miR-134/GSK3β axis, enhancing antioxidant and anti-inflammatory efficacy through nano-formulation.	(49)
Rat AD model (Aβ_25_–_35_-induced)	Synthetic compound 10c (8-hydroxyquinoline–resveratrol derivative)	Strong antioxidant effect (↓ ROS, NO, IL-1β); inhibition of microglial activation; protection of neuronal cells	10c exerted potent antioxidant and anti-inflammatory actions, improving learning and memory, suggesting a promising neuroprotective derivative of resveratrol.	(56)
Rat AD model (OVX + D-gal + Aβ_1_ –_42_)	Resveratrol 20, 40, 80 mg/kg for 12 weeks	↓ Aβ_1_ –_42_; ↓ RAGE, MMP-9, NF-κB; ↑ Claudin-5; restored BBB integrity	Resveratrol reduced neuroinflammation and preserved BBB integrity by modulating NF-κB, MMP-9, and Claudin-5, reinforcing its vascular and neuroimmune protective effects.	(10)
*In vitro* (BV2 microglia, mCRP-induced)	Resveratrol (24 h co-treatment with mCRP)	↓ NF-κB activation, ↓ NLRP3 inflammasome, ↓ COX-2, ↓ pro-inflammatory cytokines; ↑ SIRT1, ↑ Nfe2l2, ↑ Cat, ↑ Sod2	Resveratrol inhibited mCRP-induced inflammation and activated antioxidant defense genes, highlighting protection against inflammation-triggered AD risk.	(50)
Rat AD model (AlCl_3_-induced)	Oral resveratrol for 45 days (before/after AD induction)	↓ Aβ, ↓ tau, ↓ CRP, ↓ IL-6, ↓ TNF-α, ↓ TGF-β, ↓ MDA; ↑ catalase, ↑ SOD	Resveratrol exerted therapeutic and preventive effects via anti-amyloid, anti-inflammatory, and antioxidant actions.	(51)
Obesity-related AD mice	TG-peptide chitosan nanoparticles loaded with resveratrol (TG-Res-CS/TPP-NPs)	↓ Aβ, ↓ p-Tau, ↓ JNK/AKT/GSK3β signaling; ↑ GLUTs, ↑ antioxidant enzymes; regulated gut microbiota	Brain-targeted nanoformulation enhanced bioavailability, reduced insulin resistance, and modulated gut–brain axis in AD.	(52)
3xTg-AD mouse model	Resveratrol ± exercise for 5 months	↓ Aβ oligomers, ↓ apoptosis, ↓ autophagy, ↓ inflammation; ↑ BDNF, ↑ synaptic markers, ↑ SIRT1	Resveratrol and exercise synergistically attenuated neuroinflammation and Aβ toxicity, improving neuronal health.	(53)
AD mice with metabolic disorder (high-fat diet)	Resveratrol-loaded selenium/chitosan nano-flowers (Res@SeNPs@Res-CS-NPs)**	↓ LPS, ↓ JNK/AKT/GSK3β, ↓ Aβ, ↓ p-Tau; regulated Firmicutes/Bacteroidetes ratio; ↑ antioxidant enzymes	Nanoformulated Res restored gut microbiota, alleviated insulin resistance and neuroinflammation, and improved memory.	(54)
AD mice (AlCl_3_ + D-gal-induced)	Oral Res–selenium–peptide nanocomposites (TGN-Res@SeNPs)	↓ Aβ aggregation, ↓ ROS, ↓ NF-κB/MAPK/Akt; ↑ antioxidant enzymes; regulated gut microbiota (↓ Alistipes, ↓ Helicobacter)	TGN-Res@SeNPs inhibited Aβ aggregation, reduced oxidative stress and neuroinflammation, and balanced gut microbiota, enhancing neuroprotection.	(55)

AD, Alzheimer’s disease; Aβ, amyloid beta; Aβ_1_ –_42_, amyloid beta peptide 1–42; Aβ_25_–_35_, amyloid beta peptide 25–35; ADCS-ADL, Alzheimer’s Disease Cooperative Study–Activities of Daily Living; AchE, acetylcholinesterase; AlCl_3_, aluminum chloride; AMPK, AMP-activated protein kinase; BBB, blood–brain barrier; BDNF, brain-derived neurotrophic factor; Cat, catalase; CQ, clioquinol; CRP, C-reactive protein; CS, chitosan; CSF, cerebrospinal fluid; D-gal, D-galactose; ET, exercise training; FGF-2, fibroblast growth factor-2; GSK3β, glycogen synthase kinase 3 beta; IL, interleukin; IL-1β, interleukin-1 beta; IL-4, interleukin-4; IL-6, interleukin-6; IL-12p40/p70, interleukin-12 subunits p40 and p70; JNK, c-Jun N-terminal kinase; LPS, lipopolysaccharide; MDA, malondialdehyde; MDC, macrophage-derived chemokine; miR-134, microRNA-134; MMP, matrix metalloproteinase; MMSE, Mini-Mental State Examination; mCRP, monomeric C-reactive protein; mTOR, mechanistic target of rapamycin; NF-κB, nuclear factor kappa-light-chain-enhancer of activated B cells; NEP, neprilysin; NO, nitric oxide; NLRP3, NOD-, LRR-, and pyrin domain-containing protein 3; OVX, ovariectomized; pCREB, phosphorylated cAMP response-element binding protein; PI3K, phosphatidylinositol 3-kinase; PKG, protein kinase G; PKA, protein kinase A; RAGE, receptor for advanced glycation end-products; RANTES, regulated upon activation normal T cell expressed and secreted; Res, resveratrol; Resv, resveratrol; Res@SeNPs@Res-CS-NPs, resveratrol-loaded selenium/chitosan nano-flowers; RSV-SeNPs, resveratrol–selenium nanoparticles; ROS, reactive oxygen species; Se, selenium; SeNPs, selenium nanoparticles; SIRT1, sirtuin-1; SOD, superoxide dismutase; STAT3, signal transducer and activator of transcription 3; TGF-β, transforming growth factor-beta; TGN, blood–brain barrier transport peptide; TGN-Res@SeNPs, resveratrol–selenium–peptide nanocomposites; TNF-α, tumor necrosis factor-alpha; TPP, tripolyphosphate; TREM2, triggering receptor expressed on myeloid cells 2; 10c, (E)-5-(4-hydroxystyryl)quinolin-8-ol. ↓, Decrease; ↑, Increase.

Preclinical studies. To augment bioavailability and cerebral penetration, recent preclinical investigations have employed nano-formulations integrating RES with selenium (Res-SeNPs). In a rat model of AD induced by AlCl_3_, Res-SeNPs markedly mitigated oxidative stress, mitochondrial dysfunction, and cholinergic deficits, while facilitating the clearance of Aβ. Mechanistically, the intervention elevated SIRT1 expression and suppressed microRNA-134, thereby inhibiting GSK-3β-mediated tau hyperphosphorylation and STAT3/IL-1β-driven neuroinflammation. This dual modulation of oxidative and inflammatory pathways culminated in significant enhancements in neuronal morphology and cognitive performance, thereby demonstrating that selenium-based nanoparticles amplify the neuroprotective efficacy of RES (49).

Further preclinical investigations have substantiated RES’s capacity to preserve the integrity of the BBB and to attenuate inflammatory responses. In ovariectomized D-galactose-induced AD rat models, administration of RES resulted in a decrease in hippocampal Aβ1–42 accumulation and a downregulation of RAGE, MMP-9, and NF-κB expression, concurrently upregulating Claudin-5, an essential tight junction protein. These alterations not only thwarted BBB compromise but also alleviated neuroinflammatory damage linked to amyloid pathology (10). RES exhibits substantial neuroprotective properties against neurodegeneration induced by inflammatory processes. *In vitro* investigations utilizing BV2 microglia subjected to monomeric C-reactive protein (mCRP), a pro-inflammatory factor associated with post-stroke AD, demonstrated that RES significantly attenuated mCRP-mediated nitric oxide synthesis, cyclooxygenase-2 expression, and the release of pro-inflammatory cytokines. Moreover, RES inhibited the activation of the NLRP3 inflammasome and the nuclear translocation of NF-κB, concurrently promoting the expression of antioxidant enzymes (CAT, SOD2). Mechanistically, these protective actions were facilitated through the activation of SIRT1 and NFE2L2 signaling pathways, underscoring RES’s capacity to restore redox homeostasis and alleviate neuroinflammatory responses. These results underscore its potential therapeutic efficacy in impeding the progression of AD associated with chronic inflammatory conditions (50).

A comprehensive investigation was conducted to assess the therapeutic and preventive efficacy of RES in a rat model of AD induced by AlCl_3_. The presence of AD-like pathology was substantiated by elevated levels of amyloid-beta (Aβ), tau protein, AChE, CRP, IL-6, TNF-α, and TGF-β, in conjunction with biomarkers indicative of oxidative stress, such as malondialdehyde (MDA). The administration of RES, both prior to and subsequent to the induction of AD, resulted in a significant reversal of these biochemical and histopathological disturbances. Furthermore, the treatment reinstated the activities of catalase and SOD while enhancing the morphological integrity of brain tissue. The therapeutic effectiveness of RES was found to be comparable to that of the pharmacological agent Ebixa, indicating that its neuroprotective properties are facilitated through mechanisms involving both antioxidant and anti-inflammatory pathways, as well as the modulation of Aβ and tau pathology. Collectively, these findings emphasize the potential of RES to alleviate the complications associated with AD through multi-targeted molecular modulation (51).

Given the significant contribution of obesity-induced insulin resistance to the pathogenesis of AD, a recent investigation has formulated brain-targeted RES-loaded chitosan nanoparticles (TG-Res-CS/TPP-NPs) to augment their bioavailability and therapeutic efficacy. The nanoparticles were meticulously designed with a targeting peptide to enhance their ability to traverse the BBB. In a mouse model of AD that is associated with obesity, TG-Res-CS/TPP-NPs demonstrated a pronounced enhancement in cognitive performance, a reduction in tau phosphorylation and Aβ aggregation, and a restoration of insulin sensitivity via the modulation of the JNK/AKT/GSK3β signaling pathway. Furthermore, the treatment was observed to bolster antioxidant defenses, elevate the expression of glucose transporters, and diminish microglial activation. The formulation also exhibited regulatory effects on the composition of gut microbiota by reinstating beneficial bacterial populations and attenuating inflammation-associated taxa. These findings indicate that TG-Res-CS/TPP-NPs serve as a novel nanoplatform for targeted therapeutic interventions in AD by simultaneously addressing neuroinflammation and metabolic dysfunction (52).

An investigation was conducted to assess the individual and synergistic impacts of RES administration and exercise training (ET) on neurodegenerative processes in 3xTg-AD murine models. Both therapeutic approaches resulted in a reduction of neuroinflammation, accumulation of Aβ oligomers, and markers of apoptosis while simultaneously promoting synaptic plasticity and the expression of neurotrophic factors. RES exhibited a markedly greater neuroprotective effect relative to ET administered in isolation, as indicated by the upregulation of SIRT1 and neurotrophic proteins, in conjunction with the inhibition of autophagy and endolysosomal degradation pathways. The synergistic application of RES and ET did not manifest additive effects exceeding those attributable to RES alone, implying that their neuroprotective mechanisms may converge upon analogous molecular pathways. These results highlight the significance of lifestyle modifications and nutritional strategies in the modulation of AD pathology through synergistic molecular mechanisms that encompass anti-apoptotic, antioxidant, and neurotrophic signaling pathways (53).

In order to mitigate the issues surrounding the limited bioavailability of RES and the implications of metabolic disorders on AD, researchers have innovatively engineered flower-like selenium/chitosan nanoparticles (RES@SeNPs@RES-CS-NPs) to enhance delivery efficacy. In an experimental model involving mice with AD induced by a HFD, administration of RES@SeNPs@RES-CS-NPs successfully restored the equilibrium of gut microbiota, diminished levels of LPS, and mitigated neuroinflammatory responses. Moreover, this nano-formulation enhanced insulin sensitivity and lipid metabolism by effectively modulating the ratios of Firmicutes to Bacteroidetes, subsequently thwarting Aβ aggregation and tau phosphorylation via the JNK/AKT/GSK3β signaling pathway. Additionally, bacterial populations associated with oxidative stress, including Rikenella and Alloprevotella, were meticulously regulated, which culminated in improved cognitive function. This investigation underscores the significance of the gut–brain–metabolic axis as a pivotal target for the application of nanoformulated RES in the prevention of cognitive deficits associated with AD (54).

In an investigation, a nanocomposite framework integrating RES, selenium nanoparticles, and a peptide designed for BBB transport (TGN) was developed to enhance bioavailability and therapeutic specificity. The oral delivery of these TGN-RES@SeNPs markedly enhanced cognitive functioning in murine models of AD by mitigating hippocampal Aβ accumulation, oxidative stress, and neuroinflammatory responses. Mechanistically, these outcomes were facilitated through the suppression of the NF-κB/MAPK/Akt signaling pathway and the promotion of antioxidant defense mechanisms. Significantly, the nanocomposites rectified dysbiosis of the gut microbiota by diminishing pro-inflammatory genera (Alistipes, Helicobacter, Rikenella, Desulfovibrio) and fostering the proliferation of advantageous bacterial populations (Faecalibaculum). In summary, the investigation illustrates that multifunctional nanoformulations of RES can offer a holistic therapeutic approach for AD by simultaneously addressing Aβ pathology, oxidative stress, inflammation, and gut dysbiosis (55). In conjunction with naturally occurring formulations, synthetic derivatives of RES are currently under investigation for their augmented antioxidant and anti-inflammatory properties. A recently identified compound, 8-hydroxyquinoline-RES derivative (10c), demonstrated a markedly superior capacity for scavenging ROS and inhibiting the production of NO and IL-1β in microglial cells when juxtaposed with native RES. In an *in vivo* context, 10c facilitated improvements in spatial learning and memory in Aβ25-35–induced AD rat models, thereby highlighting its neuroprotective efficacy. These observations indicate that structural modifications of RES have the potential to produce derivatives with enhanced pharmacological effectiveness against oxidative and inflammatory damage associated with AD (56).

The collective evidence derived from recent experimental and clinical investigations underscores RES as a highly promising multi-target therapeutic agent for AD. RES demonstrates neuroprotective properties through the modulation of critical signaling cascades, such as SIRT1, AMPK, PDE4–cAMP–CREB–BDNF, NF-κB, and NLRP3, culminating in diminished neuroinflammation, oxidative stress, amyloid accumulation, and apoptosis, while simultaneously enhancing mitochondrial and synaptic functionality. The improvement in gut microbiota composition and insulin sensitivity further corroborates its systemic advantages, particularly in obesity-related or metabolic variants of AD. Despite RES exhibiting commendable safety and tolerability profiles, its limited bioavailability poses a significant obstacle; nonetheless, innovative nanoparticle-based formulations and intranasal administration have shown enhanced cerebral targeting and therapeutic efficacy in preclinical studies. Integrative approaches combining physical exercise or metabolic regulation may augment cognitive outcomes through synergistic stimulation of neurotrophic and mitochondrial pathways. In order to facilitate the advancement of clinical translation, it is imperative that forthcoming biomarker-guided randomized trials employ refined delivery systems and patient stratification methodologies. In summary, RES emerges as a potentially significant preventive and adjunctive strategy for attenuating the neurodegenerative trajectory associated with AD.

It should be noted that the currently available clinical evidence regarding RES in AD remains limited. Most clinical trials conducted to date involve relatively small sample sizes and short intervention periods, which may restrict the strength and generalizability of their conclusions. Therefore, although the reported findings suggest potential benefits of RES in modulating neuroinflammation and disease−related biomarkers, these results should be interpreted with caution. Larger, well−designed randomized controlled trials are still required to confirm the therapeutic efficacy and long−term safety of RES in patients with AD. In this context, the majority of mechanistic insights into the anti−inflammatory and immunomodulatory actions of RES are still derived primarily from preclinical studies, including animal models and *in vitro* experiments.

## The effects of RES on the inflammation–immunity axis in PD and MS

Recent preclinical investigations collectively corroborate the neuroprotective function of RES and its analogs across a variety of experimental paradigms pertaining to PD. These studies consistently illustrate that RES manifests its beneficial effects through mechanisms that are antioxidant, anti-inflammatory, anti-apoptotic, and protective of mitochondrial integrity, thereby addressing both central and peripheral factors contributing to dopaminergic neuronal degeneration. Utilizing a zebrafish model of rotenone-induced PD, both selenium and RES whether administered individually or within a five-compound antioxidant formulation (N-acetylcysteine, quercetin, RES, selenium, and coenzyme Q10), effectively restored motor function and dopamine concentrations. The intervention resulted in an upregulation of antioxidant enzyme expression (SOD, GPx), suppression of apoptotic and autophagic pathways, and a decrease in IL-6 levels, thereby RES the synergistic anti-inflammatory and redox-regulating properties of selenium and RES. This cooperative mechanism emphasizes the promise of multi-compound therapeutic strategies that concurrently address oxidative stress and inflammation as a viable approach to the treatment of PD (57) (**Table 3**). Expanding upon this concept, a comprehensive multi-omics investigation conducted in A53T α-synuclein transgenic mice demonstrated that a RES–hydroxypropyl-β-cyclodextrin inclusion complex (RHSD) significantly enhanced motor and cognitive functions more effectively than the unmodified form of RES. By modulating the microbiota–gut–brain axis, RHSD reinstated intestinal barrier integrity, augmented the population of beneficial Lactobacillus species, and normalized anomalous microbial metabolites linked to mitochondrial dysfunction and neuroinflammation. Transcriptomic analyses further elucidated that RHSD modulated host gene expression pertinent to the regulation of dopamine receptors and calcium signaling, thereby supporting a bidirectional interaction between gut microbiota and neuronal health. These findings furnish mechanistic evidence that the enhancement of RES bioavailability contributes to systemic and central neuroprotection through metabolic and microbial reprogramming (58).

**Table 3:** Summary of key findings of resveratrol on the inflammation–immunity axis in Parkinson’s disease and multiple sclerosis.

Model/system	Intervention	Molecular/cellular mechanisms	Key findings	Reference
Zebrafish PD model (rotenone-induced)	Selenium, resveratrol, and combined antioxidant cocktail (2QRNS)	↑ Antioxidant genes (SOD, GPx), ↓ IL-6, apoptosis, and autophagy	Selenium enhanced antioxidant defenses; resveratrol reduced inflammation, together mitigating dopaminergic loss	(57)
A53T α-synuclein transgenic mice	Resveratrol-cyclodextrin inclusion complex (RHSD)	Gut microbiota modulation (↑ Lactobacillus spp.), normalization of amino acid metabolism, ↓ neuroinflammation	RHSD improved motor and gut functions via microbiota–gut–brain axis and reduced inflammatory pathways	(58)
Neuron–glia co-culture (PC12 + N9)	Trans ϵ-Viniferin ± resveratrol	↓ LPS-induced inflammation, ↓ glial activation, ↓ apoptosis	Viniferin reduced microglial-induced inflammation and protected dopaminergic neurons; potential nutraceutical therapy	(59)
Rat PD model (6-OHDA)	Resveratrol (10–40 mg/kg)	↓ COX-2, ↓ TNF-α mRNA/protein, ↓ mitochondrial damage	Resveratrol attenuated neuroinflammation and neuronal apoptosis, restoring dopaminergic integrity	(60)
MPTP-induced PD in mice	Resveratrol	↓ IL-1β, IL-6, TNF-α, ↑ SOCS-1 expression	SOCS-1 upregulation linked to reduced pro-inflammatory cytokines and microglial activation; dopaminergic neuron preservation	(61)
PD model (nanogel formulation)	Res@Se-chitosan nanogel	Enhanced BBB penetration, ↓ NF-κB & TGF-β signaling, ↓ microglial inflammation	Nanogel improved brain delivery of resveratrol, effectively reducing neuroinflammation and oxidative stress	(62)
Rotenone-induced PD in rats	Resveratrol	↓ CHOP, ↓ GRP78, ↓ caspase-3, ↓ IL-1β, ↑ GPx, ↑ Nrf2	Resveratrol reduced ER stress, oxidative stress, and neuroinflammation via Nrf2 activation	(63)
A53T α-synuclein PD mice	Resveratrol (dose-dependent)	↓ α-synuclein aggregation, ↓ neuroinflammation, ↓ oxidative stress	Reduced Lewy pathology, improved motor/cognitive function via anti-inflammatory pathways	(64)
Human MS (relapsing-remitting, RCT, n=55)	Resveratrol 500 mg/day (8 weeks)	↓ TNF-α, ↓ MDA, mild HDL improvement	Clinical evidence of anti-inflammatory and antioxidant effects in MS patients	(65)
EAE mouse model of MS	Intranasal resveratrol nanoparticles (RNs)	↑ Retinal ganglion cell survival, ↓ inflammation/demyelination	RNs improved neuroprotection at low dose, independent of local inflammation	(66)
MS patients (relapsing-remitting and primary-progressive)	Semi-vegetarian diet + supplements (vitamin D, omega-3, resveratrol, etc.)	↓ MMP-9 activation, ↑ ω-3 FA, stable vitamin D	Nutritional therapy improved inflammatory markers and patient wellbeing	(67)
Chronic EAE mice	Resveratrol (SRT501 and standard formulation)	↑ Retinal ganglion cell survival, neuroprotection without immune suppression	Resveratrol prevented neuronal loss independent of immunosuppression	(68)
Relapsing-remitting EAE model	Oral SRT501 (SIRT1 activator)	↑ SIRT1 activation, ↑ axonal density, ↓ neuronal loss	Neuroprotection via SIRT1 activation; anti-inflammatory synergy with existing therapies	(69)

PD, Parkinson’s disease; MS, multiple sclerosis; EAE, experimental autoimmune encephalomyelitis; BBB, blood–brain barrier; RHSD, resveratrol hydroxypropyl-β-cyclodextrin; RNs, resveratrol nanoparticles; Res@SeN, resveratrol–selenium–chitosan nanogel; 2QRNS, antioxidant cocktail of quercetin, resveratrol, N-acetylcysteine, selenium, and coenzyme Q10; NAC, N-acetylcysteine; CoQ10, coenzyme Q10; SOD, superoxide dismutase; GPx, glutathione peroxidase; IL, interleukin; TNF-α, tumor necrosis factor-alpha; COX-2, cyclooxygenase-2; NF-κB, nuclear factor kappa B; SOCS-1, suppressor of cytokine signaling-1; Nrf2, nuclear factor erythroid 2-related factor 2; CHOP, C/EBP homologous protein; GRP78, glucose-regulated protein 78; MMP-9, matrix metalloproteinase-9; RAGE, receptor for advanced glycation end products; TGF-β, transforming growth factor-beta; SIRT1, sirtuin 1; RGC, retinal ganglion cell; HDL-C, high-density lipoprotein cholesterol; LDL-C, low-density lipoprotein cholesterol; TG, triglycerides; MDA, malondialdehyde; SNpc, substantia nigra pars compacta; OKR, optokinetic response; α-syn, alpha-synuclein; ROS, reactive oxygen species; DA, dopaminergic; NGF, nerve growth factor; LPS, lipopolysaccharide; ER, endoplasmic reticulum; FA, fatty acids. ↓, Decrease; ↑, Increase.

Complementing these observations, cellular investigations have demonstrated that trans-ϵ-viniferin, a naturally occurring dimer of RES, exhibits significant anti-apoptotic and anti-inflammatory properties within a neuron–glia co-culture paradigm. The interaction of viniferin with RES has been shown to safeguard dopaminergic PC12 cells against cytotoxicity induced by 6-hydroxydopamine (6-OHDA) and to alleviate microglia-mediated inflammation in N9 co-cultures subjected to LPS exposure. These effects are ascribed to the inhibition of pro-inflammatory cytokine synthesis and the maintenance of neuronal viability, implying that viniferin may enhance or complement the effects of monomeric RES in the preservation of neuron–glia homeostasis. Such findings underscore the significance of addressing both neuronal resilience and microglial activation in the context of PD pathogenesis (59). In an *in vivo* model utilizing 6-OHDA in rats, the chronic administration of RES via oral route (10–40 mg/kg over a duration of 10 weeks) resulted in a statistically significant reduction in rotational asymmetry, enhancement of dopaminergic neuronal morphology, as well as a mitigation of mitochondrial damage and chromatin condensation within the substantia nigra. Molecular investigations demonstrated a downregulation of COX-2 and TNF-α, thereby indicating a suppression of neuroinflammatory signaling pathways. The observed histological and biochemical enhancements corresponded with improved behavioral outcomes, thereby establishing RES’s dual role in the preservation of mitochondrial integrity and the inhibition of inflammatory pathways, which are pivotal contributors to the loss of dopaminergic neurons (60).

Consistent with these results, studies in an MPTP-induced mouse model of PD further confirmed RES’s anti-inflammatory and neuroprotective actions. Treatment decreased glial activation and lowered expression of IL-1β, IL-6, and TNF-α, along with their receptors, in the substantia nigra pars compacta. Importantly, RES upregulated the suppressor of cytokine signaling-1 (SOCS-1), a key regulator of cytokine signaling through the JAK/STAT pathway, providing a mechanistic explanation for its immunomodulatory effects. This was accompanied by preservation of tyrosine hydroxylase (TH) immunoreactivity, reflecting protection of dopaminergic neurons (61).

In a nanotechnology-oriented methodology, a RES–selenium–chitosan nanogel (RES@SeN) was engineered to address the challenges associated with BBB permeability and the regulation of inflammation in PD. This multifunctional construct capitalized on the modulation of the protein corona, thereby facilitating effective traversal of the BBB and subsequent accumulation within the CNS. Mechanistically, RES@SeN mitigated oxidative stress and inhibited microglial activation while promoting the anti-inflammatory M2 phenotype. Transcriptomic analyses revealed alterations in the NF-κB and TGF-β signaling pathways, both of which are critical mediators of neuroinflammation and tissue regeneration. These results underscore the translational viability of nanogel-mediated delivery systems for resve RES ratrol, offering sustained release, enhanced targeting, and improved therapeutic efficacy in the management of PD (62). In a complementary manner, an additional investigation elucidated that RES alleviates apoptosis associated with endoplasmic reticulum (ER) stress in a rotenone-induced PD rat model. RES was observed to downregulate CHOP and GRP78, pivotal markers of ER stress, concomitantly diminishing caspase-3 activity and protein oxidation. It reinstated redox equilibrium through the activation of glutathione peroxidase and the Nrf2 antioxidant signaling pathway, thereby reinforcing its bifunctional role in the attenuation of ER stress and the restoration of redox homeostasis. This investigation underscores the potential of RES to safeguard dopaminergic neurons by intervening at various intracellular stress regulatory points (63).

In a genetic model of PD (A53T α-synuclein mice), the administration of RES demonstrated a significant amelioration of both motor and cognitive impairments in a manner contingent upon dosage. The therapeutic intervention effectively inhibited the aggregation of α-synuclein, diminished oligomeric toxicity, and mitigated oxidative and inflammatory stress, thereby enhancing behavioral outcomes. The observed reduction in Lewy-like pathology further substantiates the potential of RES as a promising disease-modifying agent for synucleinopathies, thereby extending its applicability beyond PD to encompass other neurodegenerative disorders (64).

While the preceding studies highlight the neuroprotective and anti−inflammatory effects of RES in predominantly neurodegenerative conditions such as PD, its therapeutic relevance may also extend to disorders in which immune dysregulation is a central pathogenic driver. In PD, RES has mainly been investigated for its ability to mitigate oxidative stress, regulate microglial activation, and support neuronal survival. In contrast, MS is primarily characterized by autoimmune−mediated inflammation, demyelination, and infiltration of peripheral immune cells into the CNS. In this setting, the potential benefits of RES are more closely linked to its immunomodulatory properties, including the regulation of T−cell responses, cytokine signaling, and inflammatory pathways. This distinction provides an important framework for interpreting the emerging evidence on RES in MS.

A randomized, double-blind clinical investigation involving 55 subjects diagnosed with relapsing-remitting MS illustrated that RES supplementation (500 mg/day for a duration of 8 weeks) markedly diminished levels of TNF-α and MDA, which are pivotal indicators of inflammation and oxidative stress. While metabolic parameters such as fasting glucose and HDL exhibited only minor alterations, the comprehensive anti-inflammatory and antioxidant effects suggest a substantial systemic advantage, indicating that RES may augment standard MS treatment modalities via metabolic and redox modulation (65).

In a murine model of MS characterized by experimental autoimmune encephalomyelitis (EAE), the administration of RES nanoparticles (RNs) via the intranasal route demonstrated markedly superior neuroprotective effects in comparison to oral administration. The application of RNs significantly augmented the survival rates of retinal ganglion cells, alleviated demyelination of the optic nerve, and exhibited therapeutic efficacy at merely half the dosage required for oral delivery, thereby reducing systemic adverse effects. These findings underscore the significance of employing nanoparticle and intranasal delivery methodologies in achieving targeted CNS bioavailability while maintaining neuroprotective efficacy (66). Further corroborating the hypothesis of nutritional modulation of inflammatory processes, a seven-month dietary intervention trial involving subjects diagnosed with relapsing-remitting and primary-progressive MS incorporated a semi-vegetarian, calorically-restricted regimen alongside supplementation with vitamin D, omega-3 fatty acids, lipoic acid, and RES. Notably, despite the absence of alterations in vitamin D concentrations, there were substantial reductions exceeding 50% in the activated isoforms of MMP-9, indicative of a potential mitigation of inflammation mediated by matrix metalloproteinases. These findings highlight the synergistic effects of nutritional and supplemental interventions, inclusive of RES, in fostering an anti-inflammatory equilibrium and enhancing the overall well-being of patients (67).

In a chronic EAE murine model, oral administration of pharmaceutical-grade RES (SRT501) resulted in a postponement of disease manifestation, preservation of retinal ganglion cell integrity, and mitigation of neuronal degeneration without inducing immunosuppression. This observed dissociation between neuroprotective effects and immunosuppressive actions implies that RES exerts its influence directly on neuronal survival mechanisms, potentially via the activation of SIRT1 and regulation of mitochondrial function. The neuroprotective properties of RES were further supported by evidence indicating that its advantageous effects in chronic demyelinating disease models are not contingent upon significant immune suppression, thereby presenting avenues for synergistic therapeutic strategies when combined with anti-inflammatory agents (68). Similarly, in the context of relapsing-remitting EAE, the administration of oral SRT501 was observed to mitigate neuronal damage during episodes of optic neuritis, preserve axonal density within the spinal cord, and enhance neurological function. The neuroprotective effects were facilitated through the activation of SIRT1; conversely, the inhibition of this signaling pathway, achieved through the application of sirtinol, completely negated the observed neuroprotection, thereby substantiating SIRT1-dependent mechanisms. Concomitant findings with an alternative SIRT1 activator, SRT1720, further corroborated the pivotal role of this pathway in promoting mitochondrial and neuronal resilience (69). Ultimately, the evidence derived from these investigations underscores the considerable neuroprotective efficacy of RES across a range of experimental paradigms pertaining to AD and PD. RES manifests its advantageous effects predominantly via mechanisms that are antioxidant, anti-inflammatory, anti-apoptotic, and protective of mitochondrial function. It activates critical signaling cascades, including SIRT1/AMPK, Nrf2, and cAMP-CREB-BDNF, which culminate in enhanced neuronal survival, augmented synaptic plasticity, and improved cognitive performance. Furthermore, RES modulates the signaling pathways of PDE4, neprilysin, and estradiol, thereby mitigating Aβ accumulation and facilitating its degradation. From a clinical standpoint, these findings imply that RES, particularly when formulated in nanoform or in conjunction with other bioactive agents, can traverse the BBB with greater efficacy, thereby enhancing both bioavailability and therapeutic efficacy. Its capacity to modulate neuroinflammatory cytokines, oxidative stress indicators, and neurotrophic factors positions it as a highly promising adjunctive approach for early intervention or prevention in the realm of neurodegenerative disorders. However, translation to clinical use requires careful evaluation of dose, formulation, and long-term safety. Collectively, the data support the notion that RES may offer a multi-targeted, safe, and natural approach to preserve brain function and delay cognitive decline in neurodegenerative diseases.

experimental studies suggest that RES may exert neuroprotective effects in PD models through mechanisms including attenuation of oxidative stress, modulation of mitochondrial function, and suppression of neuroinflammatory pathways. Nevertheless, most of the available evidence arises from cellular and animal models, and clinical investigations remain relatively limited. As a result, the extent to which these preclinical neuroprotective mechanisms translate into clinically significant outcomes in patients with PD remains uncertain and warrants further investigation.

## The effects of RES on the ischemia-induced brain damage

Ischemic stroke occurs when cerebral blood flow is interrupted, depriving brain tissue of oxygen and essential nutrients and ultimately leading to neuronal injury and infarction. Current treatment approaches mainly focus on restoring blood flow as quickly as possible through intravenous thrombolysis or mechanical thrombectomy (70). Ischemic stroke remains one of the most serious neurological disorders worldwide and is responsible for the majority of stroke cases, accounting for nearly 87% of all reported events (71). It is a leading contributor to death and long−term disability and places a considerable economic and social burden on healthcare systems and affected families. Survivors often experience persistent complications such as epilepsy, chronic pain, depression, and cognitive impairment, which further increase long−term healthcare costs and negatively affect quality of life (72). These challenges highlight the need for more effective therapeutic strategies that can improve both survival and neurological recovery.

It was reported that RES may protect neural tissue during cerebral ischemia by modulating oxidative stress, suppressing inflammatory signaling, and regulating pathways involved in neuronal survival and apoptosis (73, 74).

In the following section, we summarize the available experimental evidence investigating the protective effects of RES in models of cerebral ischemia and discuss the molecular mechanisms that may underlie its neuroprotective actions.

A recent systematic review and meta−analysis investigated the neuroprotective effects of RES in experimental models of cerebral ischemia/reperfusion (I/R) injury using rats (75). The analysis included 41 *in vivo* studies that evaluated the impact of RES administration on neurological outcomes and biochemical markers following experimentally induced stroke (75). Across these studies, RES treatment consistently demonstrated neuroprotective effects compared with untreated control groups.

Quantitative analysis revealed that RES significantly reduced cerebral infarct volume, one of the most widely used indicators of ischemic brain damage. In addition, RES markedly improved neurological deficit scores in treated animals, suggesting a functional recovery that paralleled the structural protection observed in brain tissue. Several studies also reported a reduction in brain edema, measured as decreased brain water content, indicating that RES may help limit secondary injury processes associated with post−ischemic inflammation and blood–brain barrier disruption (75).

This meta−analysis further examined oxidative stress markers to better understand the mechanisms underlying RES−mediated neuroprotection. Treatment with RES significantly reduced levels of MDA, a key biomarker of lipid peroxidation and oxidative damage in neuronal tissue. At the same time, RES tended to increase the activity of endogenous antioxidant enzymes such as SOD, although the magnitude of this increase varied between studies and did not always reach statistical significance. These findings support the hypothesis that the antioxidant properties of RES contribute substantially to its neuroprotective effects in ischemic injury (75).

Subgroup and dose–response analyses provided additional insights into the optimal experimental conditions for RES efficacy. Among the different dosing regimens reported, a dose of approximately 30 mg/kg appeared to produce the most consistent protective effects in terms of reducing infarct volume and improving neurological outcomes (75). Moreover, studies using shorter ischemic occlusion durations—particularly around 60 minutes of middle cerebral artery occlusion (MCAO)—tended to demonstrate stronger neuroprotective effects compared with models involving longer occlusion periods. These observations suggest that both dosage and ischemia duration may influence the magnitude of the therapeutic response (75).

Despite the promising findings, the authors emphasized several limitations of the available evidence. Many of the included studies had relatively small sample sizes and limited methodological rigor, including insufficient reporting of randomization, blinding, or allocation concealment (75). In addition, statistical analyses indicated the presence of potential publication bias, suggesting that studies reporting negative or neutral results may be underrepresented in the literature. Consequently, while the aggregated data strongly support a neuroprotective role for RES in experimental cerebral ischemia, the authors concluded that further well−designed preclinical studies are necessary to clarify optimal dosing strategies, therapeutic time windows, and mechanisms of action before these findings can be reliably translated into clinical research (75).

Increased oxidative stress is widely implicated in the cascade of delayed neuronal cell death (DND) that occurs after cerebral ischemic insult (76). To test whether RES can mitigate oxidative-stress–driven neurodegeneration, a study evaluated its ability to reduce neuronal death following transient global cerebral ischemia in Mongolian gerbils. Mongolian gerbils were randomly assigned to three experimental groups: (i) sham control, (ii) ischemia, and (iii) ischemia treated with RES. Transient global cerebral ischemia was induced by occluding both common carotid arteries (CCA) for 5 minutes. Cerebral perfusion was monitored before and during the occlusion using a laser Doppler flowmeter, confirming the ischemic challenge during the CCA blockade (76).

RES was administered intraperitoneally at a dose of 30 mg/kg body weight. Importantly, the dosing regimen included two clinically relevant timing strategies: RES was given either during CCA occlusion or shortly after it, and it was administered again 24 hours after ischemia (76). This design allowed the authors to test whether RES’s protective effects depend on the timing of administration (76).

To assess neurodegeneration and neuroinflammation, brain sections were immunostained to quantify neuronal loss, as well as glial responses involving astrocytes and microglial cells. In parallel, a separate time-course study was performed to evaluate RES bioavailability in peripheral and central compartments. Specifically, serum, liver, and brain RES levels were measured by high-performance liquid chromatography (HPLC) following i.p. injection.

The mechanisms were further supported by histological findings: neuronal death was accompanied by increased reactive astrocyte and microglial activation, indicating robust neuroinflammatory signaling in the ischemic hippocampus. When RES was administered either during or after CCA occlusion, DND was significantly reduced (P<0.05), alongside a marked attenuation of glial cell activation. In other words, RES not only limited the extent of neuron loss but also dampened the inflammatory/microglial and reactive astrocyte responses associated with delayed injury progression (76).

Pharmacokinetic evidence strengthened the mechanistic plausibility of the observed protection. After i.p. injection, RES was detected in serum, liver, and brain, demonstrating that it reaches both peripheral tissues and the CNS. The HPLC time-course revealed peak levels at distinct time points across compartments: 1 hour in serum, 4 hours in the liver, and 4 hours in the brain. This temporal pattern supports systemic absorption followed by subsequent central penetration (76).

Overall, the study concludes that RES can cross the blood–brain barrier and exert protective effects against cerebral ischemic injury. By reducing DND in hippocampal CA1 and decreasing astrocytic/microglial activation, the findings provide experimental support for RES’s potential neuroprotective role in ischemia-induced oxidative stress and secondary inflammatory cascades (76).

In another study, RES’s potential neuroprotective effects against cerebral ischemia–induced hippocampal neuronal damage were investigated in an experimental rat model (77). Sixty adult male rats were anesthetized with urethane (1.4 g/kg, intraperitoneally) and divided into three groups: a sham-operated group, an ischemia group, and an ischemia group treated with RES. Cerebral ischemia was induced by bilateral ligation of the carotid arteries. In the treatment group, RES was administered intravenously at a single dose of 20 mg/kg (77).

To explore the mechanisms underlying its effects, researchers measured oxidative stress and NO levels in the hippocampus. Hydroxyl radical production was assessed indirectly by quantifying dihydroxybenzoic acid (DHBA) using microdialysis combined with high−performance liquid chromatography, while NO levels were also analyzed across the experimental groups. The results showed that cerebral ischemia significantly increased hydroxyl radical formation and led to marked neuronal loss in the hippocampus. However, rats that received RES exhibited a clear reduction in oxidative stress markers, reflected by decreased DHBA levels. At the same time, RES treatment significantly increased NO levels, which was associated with improved cerebral blood flow. These changes corresponded with a reduction in neuronal damage compared with untreated ischemic animals (77).

Overall, the findings indicate that a single administration of RES can protect hippocampal neurons from ischemia−induced injury. This protective effect appears to be mediated through its antioxidant activity, particularly by scavenging free radicals, as well as by enhancing cerebral blood flow through NO–related mechanisms (77).

Oxidative stress and free radical production play a major role in secondary neuronal damage following cerebral ischemia–reperfusion (I/R). A rat model of transient cerebral ischemia was established using MCAO, a widely used experimental model that mimics human ischemic stroke (78). RES was administered intravenously at a dose of 10^-7^ g/kg at two time points: 15 minutes before the induction of ischemia and again at the onset of reperfusion, which occurred 2 hours after arterial occlusion (78). The results demonstrated that ischemia significantly disrupted mitochondrial function in the hippocampus, particularly by reducing ATP production and impairing the activity of mitochondrial respiratory chain complexes. Treatment with RES markedly restored ATP levels and improved the activity of these mitochondrial complexes, indicating improved mitochondrial energy metabolism (78). In addition, Western blot analysis revealed that RES significantly reduced the release of cytochrome c from mitochondria, suggesting suppression of apoptosis-related pathways. Further evidence of neuroprotection was observed at the molecular level. DNA electrophoresis showed a clear reduction in genomic DNA fragmentation in animals treated with RES, indicating decreased neuronal apoptosis. The expression of stress−response proteins, including heat shock protein 70 (Hsp70) and metallothionein, was elevated following ischemia and increased even further in the RES-treated group, suggesting an enhanced cellular defense response. RES also improved several biochemical markers related to oxidative stress and cellular metabolism (78). Treatment restored mitochondrial GSH levels and glucose−6−phosphate dehydrogenase activity while normalizing serum lactate dehydrogenase (LDH). At the same time, indicators of oxidative damage, such as mitochondrial lipid peroxidation, protein carbonyl formation, and intracellular hydrogen peroxide levels, were significantly reduced. These molecular and biochemical improvements were accompanied by clear functional and structural benefits. Rats treated with RES showed significantly better neurological scores compared with untreated ischemic animals. In addition, both brain infarct volume and cerebral edema were markedly reduced. Histological examination of the hippocampal CA1 region further confirmed these protective effects, revealing decreased intercellular and pericellular edema as well as reduced glial cell infiltration. Overall, the findings suggest that RES exerts strong neuroprotective effects in cerebral ischemia–reperfusion injury. By improving mitochondrial function, reducing oxidative stress, and limiting apoptotic pathways, RES contributes to the preservation of neuronal structure and function, supporting its potential therapeutic relevance in ischemic stroke (78).

Among the mechanisms contributing to brain damage after stroke, MMP−9 has been identified as an important mediator of neuronal injury during the reperfusion phase. Excessive MMP−9 activity is associated with blood–brain barrier disruption, inflammation, and neuronal degeneration. In this study, the potential neuroprotective role of trans−RES, a naturally occurring polyphenolic compound, was evaluated with particular focus on its effects on MMP−9 activity during cerebral ischemia–reperfusion (79).

Male Balb/C mice were orally treated with RES at a dose of 50 mg/kg for seven consecutive days. After the treatment period, focal cerebral ischemia (FCI) was induced using the MCAO model, where an intraluminal filament was used to block the artery for 2 hours followed by reperfusion (79). This model is commonly used to reproduce the pathological events of acute ischemic stroke. Histological examination of brain tissues revealed that mice pretreated with RES showed noticeable improvements in tissue integrity compared with untreated ischemic animals. In particular, necrotic damage in vulnerable brain regions such as the cortex and basal ganglia was significantly reduced in the RES−treated group (79). To investigate the underlying mechanism, the study measured both the activity and gene expression of MMP−9 at several time points after the induction of ischemia. The results demonstrated that cerebral ischemia–reperfusion markedly increased MMP−9 expression and enzymatic activity in the brain. However, this elevation was significantly suppressed in mice that had received RES treatment. The reduction in MMP−9 levels suggests that RES may protect neurons by limiting the enzymatic processes that contribute to extracellular matrix degradation and blood–brain barrier damage during reperfusion injury (79).

Overall, the findings indicate that RES exerts neuroprotective effects in experimental stroke models. By attenuating the upregulation of MMP−9 following ischemia–reperfusion, RES may help reduce neuronal injury and tissue damage. These results highlight the potential of RES as a therapeutic candidate for limiting brain damage associated with acute ischemic stroke (79).

Previous research has suggested that RES can reduce infarct size and provide neuroprotection in models of FCI (80). Building on these findings, this study investigated whether NO signaling contributes to the protective mechanisms of RES following ischemic brain injury.In this experiment, FCI was induced in anesthetized Long−Evans rats by occluding the middle cerebral artery (MCA) for 1 hour, followed by 24 hours of reperfusion. After the occlusion period, RES was administered intravenously at doses of 0.1 or 1 μg/kg. The study then evaluated several biochemical markers associated with oxidative stress and neuronal damage, as well as the expression of enzymes involved in NO production (80).

The results showed that RES treatment significantly reduced markers of cellular injury and oxidative stress. Specifically, levels of LDH in plasma and MDA in brain tissue were decreased, indicating reduced tissue damage and lipid peroxidation. At the same time, plasma NO levels increased following RES administration (80). Further molecular analysis revealed that RES modulated the expression of nitric oxide synthase (NOS) isoforms (80). The treatment suppressed both protein and mRNA expression of inducible nitric oxide synthase, which is typically associated with inflammatory damage. In contrast, it enhanced the expression of endothelial nitric oxide synthase (eNOS), an enzyme involved in maintaining vascular function and cerebral blood flow. The expression of neuronal nitric oxide synthase remained largely unchanged (80). To confirm the role of NO signaling in these effects, the researchers used pharmacological inhibitors of NOS. Pretreatment with NG−nitro−L−arginine methyl ester, a nonselective NOS inhibitor, or L−N5−(1−iminoethyl)−ornithine, a selective eNOS inhibitor, completely abolished the protective effect of RES on infarct volume. This finding indicates that activation of the NO pathway, particularly through eNOS, is a key mechanism underlying RES−mediated neuroprotection (80).

Another study investigated whether RES reduces brain damage after I/R by suppressing inflammation mediated by the NLRP3 inflammasome and whether autophagy and the signaling protein Sirt1 are involved in this process.To examine these mechanisms, healthy male Sprague–Dawley rats were subjected to MCAO for 1 hour followed by 24 hours of reperfusion to induce cerebral I/R injury. The study measured inflammatory mediators, markers of autophagy, and neurological outcomes after treatment with RES (81). The results showed that cerebral I/R injury significantly increased the activation of the NLRP3 inflammasome and its downstream inflammatory components, including caspase−1, IL−1β, and IL−18. At the same time, autophagy activity was elevated, as indicated by changes in the LC3B−II/LC3B−I ratio and p62/SQSTM1 levels (81). Administration of RES, a known activator of Sirt1, markedly reduced NLRP3 inflammasome−related inflammation while further enhancing autophagy activity. Functionally, these molecular changes were accompanied by significant improvements in outcomes: RES treatment reduced cerebral infarct volume, decreased brain water content (indicating reduced edema), and improved neurological deficit scores (81). To determine whether autophagy was required for these protective effects, the autophagy inhibitor 3−methyladenine (3−MA) was administered through intracerebroventricular injection. Blocking autophagy with 3−MA abolished the ability of RES to suppress NLRP3 inflammasome activation, indicating that autophagy plays a critical role in mediating the anti−inflammatory effects of RES. The role of Sirt1 was further examined using siRNA−mediated knockdown. Suppression of Sirt1 significantly inhibited the RES−induced increase in autophagy and prevented the reduction of NLRP3 inflammasome activation. These findings suggest that Sirt1 acts upstream in the signaling pathway that links RES to autophagy activation and inflammasome suppression (81).

Preclinical studies consistently indicate that RES may confer neuroprotection in experimental models of cerebral ischemia through antioxidant, anti-inflammatory, and anti-apoptotic mechanisms. However, translation of these findings into clinical practice remains challenging. Human studies examining RES supplementation in cerebrovascular conditions are scarce, and differences in dosing, pharmacokinetics, and patient characteristics may influence therapeutic outcomes. Therefore, while experimental evidence supports the potential of RES as a neuroprotective agent, further clinical research is necessary to clarify its efficacy in human stroke populations.

## Limitations

Although a multitude of experimental and clinical investigations substantiate the neuroprotective properties of RES, several limitations must be acknowledged when evaluating the extant evidence. First, the preponderance of available data stems from *in vitro* and animal model studies, which may not adequately replicate the intricate pathophysiology of human neurological disorders. The translational gaps that exist between preclinical and clinical research remain considerable, particularly in relation to dosage, routes of administration, and duration of treatment. Second, the limited bioavailability and rapid metabolism of RES significantly impede its therapeutic efficacy. Following oral administration, RES undergoes extensive first-pass metabolism in the liver and intestine, culminating in the production of inactive metabolites and diminished systemic concentrations. These pharmacokinetic constraints render it challenging to attain effective concentrations within the brain, where the neuroprotective effects of RES are anticipated to manifest. Third, the heterogeneity present among clinical trials, encompassing variances in study design, characteristics of the population, stages of the disease, biomarkers evaluated, and formulations of RES, complicates the interpretation and comparative analysis of the findings. Discrepancies in dosing regimens and the absence of standardized outcome measures further constrain the reproducibility of results and obstruct the establishment of optimal therapeutic protocols. Fourth, data pertaining to long-term safety and efficacy remain insufficient. The majority of clinical investigations are characterized by brief follow-up durations and limited sample sizes, thereby precluding definitive conclusions regarding the sustained neuroprotective advantages of RES and the potential adverse effects associated with chronic administration. Furthermore, the interactions between RES and frequently prescribed pharmacological agents in neurological patients continue to be inadequately explored. Finally, the intricate interplay among inflammatory, immune, and memory-related pathways in neurodegenerative disorders presents a significant challenge for mechanistic interpretation. It remains ambiguous whether the advantageous effects of RES are predominantly influenced by direct neuronal modulation, systemic anti-inflammatory mechanisms, or auxiliary immunoregulatory processes. Subsequent investigations ought to rectify these inadequacies via extensive, rigorously controlled clinical trials, the innovation of enhanced formulations or delivery methodologies (e.g., nanoparticles, liposomes, or conjugates), and thorough mechanistic inquiries that incorporate molecular, cellular, and behavioral outcomes. Such initiatives will be paramount in substantiating the translational potential of RES as a plausible neuroprotective strategy.

In addition to the general challenges common to RES research, this review has several methodological limitations that should be acknowledged. Because it was conducted as a narrative review, it did not follow a predefined systematic search strategy, which may introduce selection bias in the identification of relevant studies. Although key databases and reference lists were consulted, the absence of explicit screening criteria and structured study selection procedures limits the transparency and reproducibility of the search process. Furthermore, the included studies were not evaluated using a formal risk−of−bias or study−quality assessment tool, making it difficult to compare methodological rigor across preclinical and clinical investigations. These constraints should be considered when interpreting the strength of the evidence summarized here and underscore the value of future systematic reviews and meta−analyses that apply standardized search protocols and quality−assessment frameworks.

Several methodological limitations of this review should also be acknowledged. First, this work was conducted as a narrative review and did not follow a formal systematic search strategy. Although multiple databases and relevant references were consulted, the absence of a predefined search protocol and screening framework may introduce selection bias in the identification and inclusion of studies related to RES. Second, the review did not apply a formal risk-of-bias or study-quality assessment tool to the included literature. As a result, differences in methodological rigor across experimental and clinical studies could not be systematically evaluated. Finally, as in many areas of experimental pharmacology, the available literature on RES may be influenced by publication bias, with studies reporting positive or mechanistically supportive outcomes being more likely to appear in the published record. These factors should be considered when interpreting the overall strength of the evidence summarized here and highlight the importance of future systematic reviews and meta-analyses that apply standardized methodological frameworks.

An important consideration when interpreting the literature on RES and other dietary polyphenols is the potential influence of publication bias and selective reporting of positive findings. Experimental studies, particularly in preclinical models, frequently report beneficial antioxidant, anti-inflammatory, or neuroprotective effects of polyphenolic compounds, whereas neutral or negative results may be underreported. This tendency can lead to an overestimation of therapeutic efficacy in the published literature. In addition, substantial heterogeneity exists among studies with respect to experimental design, dosing regimens, model systems, and outcome measures, which further complicates cross-study comparisons. Consequently, although the body of preclinical evidence supporting the neuroprotective potential of RES is extensive, the reliability and reproducibility of positive findings should be interpreted with caution until supported by larger, well-controlled, and transparently reported clinical investigations.

## Future perspectives

Future research endeavors concerning RES ought to focus on surmounting existing experimental and clinical impediments while concurrently enhancing our comprehension of its neuroprotective mechanisms. A paramount objective is the innovation of novel delivery systems that augment its bioavailability, stability, and cerebral penetration. Sophisticated technologies, including nanoparticle-based carriers, liposomal formulations, and prodrug methodologies, have the potential to markedly enhance its pharmacokinetic profile and therapeutic efficacy in addressing CNS disorders. Furthermore, forthcoming investigations should concentrate on dose-response relationships, treatment duration, and formulation-specific effects to ascertain optimal therapeutic regimens tailored for various neurological conditions. Comprehensive, randomized clinical trials featuring standardized outcome measures and extended follow-up durations are imperative to substantiate the efficacy and safety of RES as observed in preclinical studies. From a mechanistic standpoint, multi-omics methodologies encompassing genomics, proteomics, metabolomics, and transcriptomics may yield profound insights into the mechanisms by which RES influences neuronal, inflammatory, and immune signaling networks. Such integrative assessments have the potential to elucidate novel molecular targets and biomarkers linked to cognitive enhancement and neuroprotection. Furthermore, subsequent investigations should examine combinatorial strategies that incorporate RES alongside other neuroprotective compounds, antioxidants, or lifestyle modifications, including physical activity or dietary alterations. These synergistic methodologies may augment therapeutic efficacy and confer more extensive protection against neurodegenerative processes. Lastly, it is imperative to investigate the long-term neurocognitive and behavioral ramifications associated with RES supplementation, particularly in at-risk populations such as the elderly and individuals with early-stage neurodegenerative disorders. Addressing these research deficiencies will facilitate the translation of RES’s promising laboratory outcomes into effective clinical interventions aimed at preserving cognitive health and mitigating neurological deterioration.

Beyond improvements in formulation strategies and the expansion of randomized clinical trials, several unexpected observations reported in experimental studies deserve more explicit consideration, as they may provide important clues about the context−dependent nature of RES activity in the nervous system. One notable finding is the reported competitive interaction between RES administration and EE. EE is a powerful modulator of neuroplasticity, capable of enhancing hippocampal neurogenesis, synaptic remodeling, and cognitive performance through pathways that include BDNF signaling, CREB activation, and metabolic regulation. The observation that RES does not necessarily produce additive benefits in the presence of enrichment suggests that both interventions may converge on overlapping molecular pathways. This raises the possibility that the magnitude of RES−mediated neuroprotection could depend on baseline levels of neuronal stimulation, lifestyle factors, or environmental conditions. Future studies should therefore examine how behavioral context—such as physical activity, cognitive stimulation, and environmental complexity—modulates the efficacy of RES, both in animal models and in human populations. Such work may help clarify whether RES acts primarily as a compensatory modulator under conditions of reduced neuroplastic stimulation or whether its benefits are attenuated when endogenous neurotrophic pathways are already strongly activated.

Another observation that merits closer examination is the mild paradoxical elevation of certain cytokines reported in the AβPP/PS1 model (**Table 1**). Although RES is widely characterized as an anti−inflammatory compound, this finding suggests that its effects on neuroimmune signaling may be more nuanced and temporally dynamic. Rather than producing uniform suppression of inflammatory mediators, RES may induce a controlled immunomodulatory response that involves transient activation of specific cytokine pathways associated with immune remodeling, microglial phenotype switching, or tissue repair processes. Similar patterns have been described for other agents that promote resolution of inflammation rather than simple inhibition of immune activity. In this context, modest increases in selected cytokines may reflect a shift toward adaptive or regulatory immune states rather than detrimental neuroinflammation. Clarifying this issue will require longitudinal analyses of cytokine profiles, microglial activation states, and downstream signaling pathways across different stages of disease progression.

These findings collectively highlight the importance of moving beyond a simplified “antioxidant/anti−inflammatory” framework toward a more integrated understanding of RES as a context−dependent modulator of neuroplastic and neuroimmune networks. Future research should therefore prioritize experimental designs capable of capturing these interactions, including studies that combine pharmacological interventions with controlled environmental manipulations, as well as longitudinal immune and transcriptomic profiling. Such approaches may help explain the variability observed across experimental and clinical studies and could ultimately inform more precise strategies for integrating RES−based interventions with lifestyle or environmental factors in neurodegenerative disease management.

An additional limitation that should be considered when interpreting the current evidence is the potential influence of publication bias in the RES literature. As is common in rapidly expanding experimental fields, studies reporting positive or mechanistically supportive findings may be more likely to be published than those reporting neutral or negative outcomes. This tendency may contribute to an overrepresentation of beneficial effects of RES in preclinical models and could partly explain the discrepancy between robust experimental results and the more modest or variable outcomes observed in clinical studies. Greater transparency in reporting negative findings, the publication of replication studies, and the use of preregistered experimental protocols may therefore be important steps toward generating a more balanced and reliable evidence base for evaluating the therapeutic potential of RES in neurodegenerative diseases.

## Conclusions

RES, a naturally occurring polyphenolic compound present in grapes, berries, and peanuts, has garnered attention as a potentially effective neuroprotective agent owing to its diverse biological activities. Taken together, the evidence summarized across AD, PD, and MS indicates that RES (RES) and its derivatives exert broad neuroprotective actions primarily through modulation of oxidative stress, neuroinflammation, mitochondrial quality control, and synaptic plasticity. Yet the magnitude and certainty of these effects vary substantially across disease contexts and study designs, with robust preclinical data contrasted by relatively modest and heterogeneous clinical findings. In AD models, RES consistently enhances learning and memory, reduces oxidative injury, and improves neuronal survival through activation of SIRT1/AMPK and BDNF–CREB signaling, suppression of Aβ and tau pathology, and restoration of autophagy–mitophagy equilibrium. Metabolites such as dihydro−RES further strengthen these effects via NLRP3 inflammasome inhibition and mitochondrial homeostasis maintenance. These mechanistic findings are well replicated in experimental systems and underlie the rationale for clinical translation.

In clinical AD trials, long−term high−dose oral RES (up to 1 g twice daily) produces measurable shifts in cerebrospinal fluid biomarkers: decreases in MMP−9, TREM2, and neuronal injury markers, and increases in IL−4, FGF−2, and MDC—collectively denoting a possible transition from pro−inflammatory to adaptive immune signaling. While these biomarker changes are biologically meaningful, improvements in cognitive outcomes remain limited and inconsistent, reflecting challenges in sample size, dosing, and bioavailability. For PD and MS, data remain predominately preclinical but reveal coherent mechanistic patterns: antioxidative and anti−inflammatory effects, preservation of dopaminergic or oligodendroglial integrity, attenuation of mitochondrial dysfunction, and potential regulation of the gut–brain axis in PD through microbiota and barrier modulation. These findings reinforce the notion of RES as a multitarget modulator rather than a single−pathway drug. Methodologically, preclinical studies typically use uniform animal models and sustained RES dosing (10–40 mg/kg) that yield clear biochemical and behavioral improvements but limit generalizability to human disease. Clinical trials, although longer and higher in dose (up to 2 g/day), face constraints of poor systemic and CNS exposure, high inter−individual variability, and small sample sizes. Across all disease contexts, the gap between mechanistic promise and clinical robustness remains substantial.

Overall, RES emerges as a pleiotropic, multi−pathway modulator of neuroinflammatory and oxidative signaling with promising translational implications. However, its current status should be framed as a potential adjunctive therapy under experimental evaluation, not a proven disease−modifying agent. Future work must emphasize bioavailability−enhanced formulations, standardized dosing protocols, and large, well−controlled randomized trials that include both biomarker and clinical endpoints. Importantly, the present body of evidence still lacks critical components of the “personalized” framework initially proposed. No systematic effort has been made to assess patient stratification—for example, identifying subgroups based on genetic background, inflammatory profile, or metabolic capacity that may differentially respond to RES. Similarly, sex−based differences in RES absorption, metabolism, and sirtuin pathway activation remain unexplored, despite evidence from pharmacokinetic studies indicating sex−specific variations in glucuronidation and sulfation patterns. Likewise, future clinical trials should incorporate biomarker−guided designs, using molecular and imaging indicators (e.g., SIRT1 activity, oxidative stress markers, CSF cytokine panels) to tailor dosing, predict responsiveness, and monitor longitudinal efficacy. Integration of these personalized elements will be essential to move RES from a broad antioxidant candidate toward a precision−guided neuroprotective strategy. Until such individualized data and optimized trials are available, the therapeutic application of RES in neurodegenerative diseases should be pursued with cautious optimism—recognizing its extensive mechanistic reach, limited clinical validation, and untapped potential in stratified and biomarker−driven patient populations.
